# A novel hybrid optimization enabled robust CNN algorithm for an IoT network intrusion detection approach

**DOI:** 10.1371/journal.pone.0278493

**Published:** 2022-12-01

**Authors:** Ahmed Bahaa, Abdalla Sayed, Laila Elfangary, Hanan Fahmy

**Affiliations:** 1 Faculty of Computers and Artificial Intelligence, Department of Information Systems, Helwan University, Helwan, Egypt; 2 Faculty of Computers and Artificial Intelligence, Department of Information Systems, Beni-Suef University, Beni Suef, Egypt; Hanyang University, REPUBLIC OF KOREA

## Abstract

Due to the huge number of connected Internet of Things (IoT) devices within a network, denial of service and flooding attacks on networks are on the rise. IoT devices are disrupted and denied service because of these attacks. In this study, we proposed a novel hybrid meta-heuristic adaptive particle swarm optimization–whale optimizer algorithm (APSO-WOA) for optimization of the hyperparameters of a convolutional neural network (APSO-WOA-CNN). The APSO–WOA optimization algorithm’s fitness value is defined as the validation set’s cross-entropy loss function during CNN model training. In this study, we compare our optimization algorithm with other optimization algorithms, such as the APSO algorithm, for optimization of the hyperparameters of CNN. In model training, the APSO–WOA–CNN algorithm achieved the best performance compared to the FNN algorithm, which used manual parameter settings. We evaluated the APSO–WOA–CNN algorithm against APSO–CNN, SVM, and FNN. The simulation results suggest that APSO–WOA–CNf[N is effective and can reliably detect multi-type IoT network attacks. The results show that the APSO–WOA–CNN algorithm improves accuracy by 1.25%, average precision by 1%, the kappa coefficient by 11%, Hamming loss by 1.2%, and the Jaccard similarity coefficient by 2%, as compared to the APSO–CNN algorithm, and the APSO–CNN algorithm achieves the best performance, as compared to other algorithms.

## 1. Introduction

The Internet of Things (IoT), cloud computing, mobile technology, and artificial intelligence will play a significant role in Klaus Schwab’s Fourth Industrial Revolution, which will make our society more intelligent [1–3]. The IoT is a technology that is growing quickly, with fifty billion devices expected to be in use worldwide [4, 5]. IoT devices have become easy targets for attackers because they do not have basic security protocols [6, 7]. According to Statistica [8, 9], 5200 attacks are launched against IoT devices monthly. Following the spread of COVID-19, it was found that 71% of security specialists believe that security vulnerabilities in IoT networks are increasing [10, 11]. Some studies [12–14] describe the attacks and security vulnerabilities of each IoT layer and suggest ways to protect privacy [15–17]. Attacks on Internet of Things devices have increased and several studies have developed intrusion detection systems [18–24].

An intrusion detection system (IDS) scans networks or systems for malicious activity and policy violations and generates alerts and reports of malicious activity [25, 26]. Multiple systems are able to conceal suspicious network activity, hence an intelligent IDS is required. Network intrusion detection system (NIDS) and host intrusion detection system (HIDS) are two main types of IDS [27].

Several researchers have used the same botnet virus N-BaIoT dataset as in this study to examine their own techniques. Bahsi et al. [28] used feature selection to reduce the total number of features utilized in the IoT bot detection process and this resulted in higher accuracy rates. Meidan et al. [29] extracted statistical characteristics that define normal IoT traffic and trained a deep autoencoder to identify DDoS attacks. According to the experimental results, the anomaly detection techniques may significantly enhance the detection accuracy of attack traffic.

In fact, as shown in [30–32], machine learning (ML) technologies are used in intrusion detection systems, but dataset pre-processing and anomaly traffic detection are time-consuming and complex [33, 34]. However, using deep learning (DL) technologies [35–38], features may be mapped to a higher degree with more distinct feature spaces. Combining DL technologies and a traditional ML model reduces the preprocessing time [39]. Support vector machines (SVM), k-nearest neighbors (KNN), naïve Bayes, and other algorithms were used to detect IoT attacks on networks, decrease the false alarm rate, and improve the detection rate [40–46]. A CNN is a deep learning approach that uses a convolution layer to automatically extract useful features from the authentic data feature plane through the convolution layer.

The hyperparameters should be tuned throughout the neural network training and weight learning operations. For this reason, several researchers used adadelta, rmsprop, adamax, adagrad, and Adam for tuning hyperparameters [47–49]. There are several hyperparameters that should be tuned for neural network structure, including the pooling layer, hidden layers, convolutional layer, and the number of neurons in the full connected layer.

To improve a CNN’s adaptability when the hyperparameters are unknown, it is prudent to conceive of unknown hyperparameter optimization techniques. Biological heuristic techniques, such as the ant colony algorithm, genetic algorithm, artificial bee colony algorithm, PSO algorithm, whale optimization algorithm, and biogeography-based optimization algorithm, improve the performance of deep learning models [50–53]. The WOA algorithm mimics the behavior of humpback whales, using a collection of random candidate solutions, and three rules are utilized to update and enhance the position’s candidate solutions [54]. The PSO algorithm is an evolutionary algorithm based on the behavior of birds when hunting [55]. The APSO algorithm improves the PSO algorithm by changing the method of calculating the inertial weight factor [56].

In this research, the researchers propose a neural network-based optimized method adaptive particle swarm optimization–whale optimizer algorithm (APSO-WOA) for convolutional neural network (APSO-WOA-CNN) to identify hyperparameters for detecting various network attacks. The following is a summary of the novel aspects of this research:

1. A novel hybrid meta-heuristic APSO-WOA for optimization of the hyperparameters of a convolutional neural network (APSO-WOA-CNN) is presented.
2. We compare our proposed optimization algorithm with others, such as the APSO algorithm, for optimization of the hyperparameters of a convolutional neural network.
3. We evaluate the APSO–WOA–CNN algorithm against SVM, APSO–CNN, and feedforward neural network (FNN) using five evaluation indicators.

The remainder of this paper is structured in six sections as follows. Section 2 discusses the literature review. Section 3 discusses datasets and data preprocessing techniques for detecting network attacks. Section 3 discusses the framework of the 1D CNN algorithm and APSO–WOA and APSO optimization algorithms. Section 4 discusses the experiment that depends on the APSO–WOA–CNN and APSO–CNN algorithms. Section 5 discusses the results and performance analysis, based on the APSO–WOA–CNN, APSO–CNN, SVM, and FNN algorithms. Section 6 summarizes the paper and provides recommendations for future work.

## 2. Literature review

Recently, researchers in the area of network security have given greater attention to intrusion detection systems due to their increasing use [57]. We have published a systematic literature review (SLR) in monitoring real time security attacks for IoT systems [45]. The aim of SLR is to identify studied used machine learning algorithms for the detection of IoT attacks. This SLR helps us to identify the gaps in machine learning algorithms for the detection of IoT attacks. We identified forty-nine primary studies depend on the quality criteria. We summarized these primary studies based on the machine learning algorithms, datasets, IoT attack types, evaluation metrics, and monitoring real time security attacks via DevSecOps pipelines. The results show that more data preprocessing techniques should be applied to enhance the quality of public datasets. Furthermore, hybrid deep learning models must be used to keep improving the performance of IoT attack detection models.

ML methods are used in intrusion detection systems, but dataset pre-processing and anomaly traffic detection are time-consuming and complex [58]. However, using DL methods [59], features may be mapped to a higher degree with more distinct feature spaces. A CNN is a deep learning approach that uses a convolution layer to automatically extract useful features from the authentic data feature plane through convolution layer. Choosing optimal hyperparameters for deep learning models is crucial [60]. There are two main kinds of techniques for optimizing hyperparameters: automatic search methods and manual search methods. The manual configuration method makes it difficult to randomly find a good network structure. It is difficult to automatically tune hyperparameters for the optimal model structure. Tuning hyperparameters is an optimization issue in which the objective function is uncertain. It is impossible to use traditional optimization approaches such as the gradient descent or Newton method [61]. Biological heuristic techniques, such as the ant colony algorithm, genetic algorithm, artificial bee colony algorithm, PSO algorithm, whale optimization algorithm, and biogeography-based optimization algorithm, improve the performance of deep learning models by hyperparameter tuning.

Caroline et al. [62] proposed hybrid optimization techniques based on particle swarm and genetic optimization to discover optimal hyperparameters for the fully connected layers. This study improves the F1-score by 0.90 for all three networks: ResNet-50, VGG-16, and DenseNet-201.

Gurukumar et al. [63] proposed the fitness sorted rider optimization algorithm (FS-ROA) to discover optimal hyperparameters such as the pooling layer, hidden layers, convolutional layer, and the number of neurons in the full connected layer. This study achieved a good recognition rate of more than ninety seven percentage.

Lee et al. [64] used parameter-setting-free harmony search (PSF-HS) to increase CNN performance via hyperparameter adjustment in feature extraction. However, to train the PSF-HS algorithms for image recognition, a host requires reasonably powerful computing resources.

Wu Chen et al. [65] used Bayesian optimization to discover the optimal hyperparameters for machine learning algorithms. According to the experiment, the proposed model can determine the optimal hyperparameters for popular machine learning models such as neural networks and the random forest algorithm.

Amirabadi et al. [66] introduced two suboptimal grid search algorithms to modify deep learning network hyperparameters for optical communication (OC) systems. However, results demonstrate a significant decrease in computational complexity.

Lokku et al. [67] introduced a CNN-based classifier for an optimal face recognition network to identify facial images affected by occlusion and high noise.

Elmasry et al. [68] proposed a model based on PSO to identify hyperparameters and feature subsets in a single process for deep learning models such as the long short-term memory recurrent neural networks (LSTM-RNN), deep belief networks (DBN), and deep neural networks (DNN). Sakr et al. [69] tuned the SVM algorithm based on smart particle swarm optimization (SPSO). Xiu Kan et al. [56] proposed the APSO convolutional neural network algorithm to identify various types of IoT network attacks.

Alharbi et al. [70] proposed a model based on local-global best bat algorithm, PSO, and bat algorithm (BA) to identify neural network hyperparameters used for IoT attack detection. The proposed LGBA-NN achieved better accuracy than BA-NN and PSO-NN.

Sajjad et al. [71] proposed a method to identify hyperparameters of ML algorithms based on the genetic algorithm (GA) and grey wolf optimization (GWO). According to experimental findings, the training phases perform better in every trial. Additionally, GWO performs better with a *p*-value of 2.6E. Andrzej et al. [72] proposed a 3D search space whale optimization algorithm that used hyperparameter optimization, but this achieved low accuracy. Ali et al. [73] proposed an evolutionary sparse convolution network IDS to identify various types of IoT network attacks. This model decreased the false alarm rate and improved the detection rate. However, this model is time-consuming and requires complex computation. Abdullah et al. [74] proposed a modified GWO to identify hyperparameters of Extreme Learning Machine. The propsed model decrease false positive rate to less than thiry presentage. Vartouni et al. [75] suggested a technique for feature learning based on deep neural networks with an isolation forest as a classifier. Fereshteh et al. [76] proposed an IDS model based on two methods: the first method used Logistic Regression (LR) for feature selection and the second method used ANN for classification. Gonzalo et al. [77] provide a system for cloud-based distributed deep learning with the goal of detecting and mitigating phishing and botnet attacks.

Unlike these research studies, we proposed a novel meta-heuristic hybrid adaptive particle swarm optimization–whale optimizer algorithm (APSO–WOA) for optimization of the hyperparameters of a convolutional neural network (APSO-WOA-CNN). In this study, we tried to find ten hyperparameters for the CNN model by using the N-BaIoT dataset.

## 3. Datasets and data preprocessing

### 3.1. Datasets

Mohammed et al. [78] created the N-BaIoT dataset, which has one hundred and fifteen features within its data samples. The ports of all IoT devices were mirrored to acquire the dataset. After setting up the network, benign data were instantly gathered to guarantee that the data comprised normal traffic. For each statistical value, the times between the packet arrivals were extracted for two kinds of packet sizes (both inbound and outbound/only inbound), packet jitters, and packet counts. Each of the five time-windows (1 min, 10 s, 100 ms, 500 ms, and 1.5 s) had twenty-three features extracted from them, with a total of one hundred and fifteen features. All one hundred and fifteen features were used in our model. According to Table 1, a total of twenty-three statistical characteristics were computed from each window.

**Table 1:** The N-BaIoT dataset’s specific features [79].

Value	Statistic
Outgoing packet size	Variance and mean
Packet count	Number
Packet jitter	Number, mean, and variance
The packet sizes of both inbound and outbound packet arrivals together	Radius, correlation coefficient, magnitude, covariance

### 3.2. Data preprocessing

The Danmini Doorbell dataset was used for the multiclassification tasks. The original dataset used in this study contained 90,000 samples, which were generated from 10,000 random records that were selected from every type of dataset. Different sample values for the same feature differed greatly, and some unusually small or large pieces of data deceived the model’s training process. Additionally, the dispersed distribution had an effect on the training outcomes. This process generated a dataset consisting of 115 characteristic dimensions. Every feature in the dataset was standardized by mutating data into a standard normal distribution with a unit variance and a mean of zero.

The generated data matrix illustrates X^nxp^, where p and n are the feature and sample numbers, respectively. The following is a description of the primary standardization process:

#### 3.2.1. Calculate each feature’s mean

$$\mu_{j}=\sum_{i=1}^{n}\;x_{i}^{j}/n$$ (1)

where n represents the total samples, μ_j_ represents each feature’s mean, j represents the feature, i represents the sample, and $x_{i}^{j}$ represents the feature of the sample.

#### 3.2.2. Calculate each feature’s variance

$$\sigma_{j}^{2}=\sum_{i=1}^{n}\;{\left(x_{i}^{j}-\mu_{j}\right)}^{2}/n$$ (2)

where $\sigma_{j}^{2}$ represents each feature’s variance.

#### 3.2.3. Calculate the feature’s standardization characteristics for each sample

$${\bar{x}}_{i}^{j}=(x_{i}^{j}-\mu_{j})/\sigma_{j}$$ (3)

where ${\bar{x}}_{i}^{j}$ represents the feature’s standardization characteristics for each sample.

Ninety-thousand records were divided into nine-thousand validation and eighty-one-thousand training samples after feature standardization.

## 4. Methodology

In this section, we discuss the framework of the one-dimensional CNN algorithm and APSO–WOA and APSO optimization algorithms.

### 4.1. CNN Algorithm (1D)

**Fig 1:**
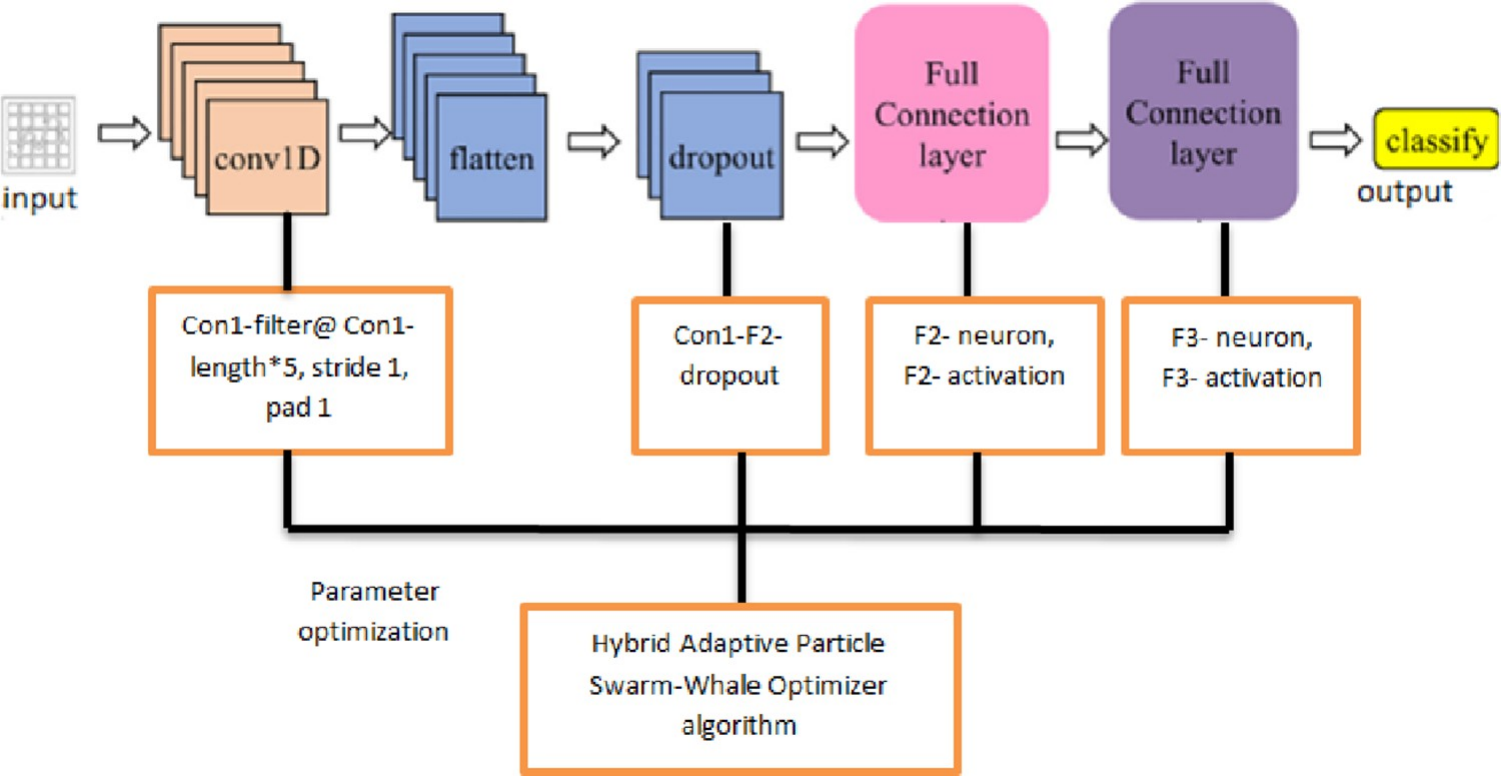
The detailed network structure model of CNN’s one-dimensional framework.

A CNN is a multi-layered neural network, and each network layer is made up of various two-dimensional planes [80]. Using the weighted total of the previous layer’s elements, each neuron’s output and activation can be calculated. In this study, we employed a 1D CNN, which was constructed using the Keras library, to identify the different types of IoT network attacks. Fig 1 illustrates the detailed network structure model of CNN’s one-dimensional framework.

#### 4.1.1. First layer

The characteristics of the N-BaIoT dataset’s attributes extract 23 statistical characteristics in five time-windows. In this study, each sample’s input dimension was allocated in the 23 × 5 feature plane. As illustrated below, the neuron’s output on the feature plane in the C1 layer was $c1_{ij}^{\mathrm{out}}$:

$$c1_{ij}^{\mathrm{out}}=F(\sum_{i=1}^{fl\times 5}\;w_{t}^{in}\times f\_raw_{t}^{in})$$ (4)

where $w_{t}^{in}$ represents the convolution kernel’s weight, fl represents the filter’s length, and $f\_raw_{t}^{in}$ represents the feature of the characteristic plane’s position. In this study, tanh, sigmoid, and relu non-linear activation functions are designated by F_i_(⋅)(i = 1,2,3), as shown below [54]:

$$F_{3}(x)=\mathrm{relu}\left(x\right)=max(0,x)$$ (5)

$$F_{2}(x)=\tanh\;\left(x\right)=\left({exp}^{x}-{exp}^{-x}\right)\left({exp}^{x}+{exp}^{-x}\right)$$ (6)

$$F_{1}(x)=sigmoid\;(x)=1/(1+{exp}^{-x})$$ (7)

The original data’s deeper and more complex features are obtained by the convolution layer. Before connecting a layer to the following layer, the feature plane is flattened as well. During the training process for a network, overfitting happens when the training set is very accurate but the validation set is not. To enhance the network’s capacity for generalization, Hinton advocated in [81] that 50% of feature detectors fail during training, also known as dropout. So, some neurons may not use in other layer, and each training cycle has a different set of neuron nodes that work. So, not all neurons working together improve network generalization.

#### 4.1.2. Second layer (F2 layer)

This layer is the first that has a full connection. In the connective layer, the neuron activation mode and number are set. In each training batch, every connective neuron is coupled to the layer below it. $full1_{m}^{\mathrm{out}}$ represents the output of the m-th neuron in the second layer, as described below:

$$full1_{m}^{\mathrm{out}}=F(\sum_{n=1}^{n_{\mathrm{keep}}}\;w_{mn}^{\mathrm{keep}}\times {\mathrm{keep}}_{n}+b_{m}^{f2})$$ (8)

where $b_{m}^{f2}$ is the neuron’s offset value in the second layer, n_keep_ is the neuron number that remains after flattening and dropout are performed, keep_n_ is the neuron used that remains from the past layer, and $w_{mn}^{\mathrm{keep}}$ is the connection weight between the neuron of the second layer and the working neuron remaining from the previous layer.

**4**.1.3. Third layer (F3 **layer)**

This layer is the second layer that has a full connection. In this layer, the neuron activation mode and number are set. All neurons in this layer are linked to the second layer neurons. The last two full connection layers enhance the learning of the flattened features.

#### 4.1.4. Fourth layer (output layer)

The third layer’s output is passed to the fourth layer, and the multi-classification task categories determine the number of fourth-layer neurons. As shown below, the softmax formula is utilized to assign the probability of every network attack in the fourth layer:

$$softmax\;(y{)}_{i}={exp}^{y_{i}}/\sum_{i=1}^{kind}\;{exp}^{y_{i}}$$ (9)

where kind is the volume of the network attack types, and softmax (y)_i_ is the i-th neuron’s output value in the fourth layer. The prediction outcomes from the training have the greatest probability. The difference between the two probability distributions for the actual and estimated values is measured by cross-entropy, as shown below:

$$H(p,q)=-\sum_{x}\;p(x)log\;q(x)$$ (10)

In this study, SGD was chosen to enhance the model’s training process to increase the detection accuracy; SGD adjusts the bias value of neurons and the connection weight.

### 4.2. Adaptive particle swarm algorithm

Eberhart and Kennedy proposed the PSO algorithm, which is an evolutionary algorithm based on the behavior of birds when hunting [55]. The method of birds searching for food is similar to that of finding optimal particle solutions. Every particle’s speed can be calculated using only the best global fitness values and the local optimal fitness value achieved in that iteration. There are two parameters for each particle, namely, the position parameter, represented by $\chi_{i}^{t}=[\chi_{i1}^{t},\chi_{i2}^{t},\chi_{i3}^{t},\ldots ,\chi_{id}^{t}]$, and the velocity parameter, represented by $v_{i}^{t}=[v_{i1}^{t},v_{i2}^{t},v_{i3}^{t},\ldots ,v_{id}^{t}]$. The formula used to update the position and velocity of each particle throughout an iteration is as follows:

$$v_{id}^{t+1}=\omega v_{id}^{t}+c_{1}r_{1}({\mathrm{pbest}}_{id}-x_{id}^{t})+c_{2}r_{2}({\mathrm{gbest}}_{d}-x_{id}^{t})$$ (11)

$$x_{id}^{t+1}=x_{id}^{t}+v_{id}^{t+1}$$ (12)

where $x_{id}^{t}$ and $v_{id}^{t}$ are the previous positions and velocity of the particle i, $x_{id}^{t+1}$ and $v_{id}^{t+1}$ are the position and velocity of the particle i during iteration t + 1, t represents the current iteration number, c_2_ and c_1_ are represented by the acceleration coefficients, pbest_id_ represents the i-th particle’s optimal position during the present iteration, w represents the inertial weight, gbest_d_ represents the population’s optimal global position, and the two random values r_1_ and r_2_ are equally distributed between zero and one.

Particles may readily escape the current optimal local value when the inertial weight is enormous and may not converge later. When the inertial weight is in an optimal state, particles fall towards the optimal local value. Consequently, the inertial weight must be adjusted dynamically. An algorithm called APSO for dynamic inertial weight was developed based on these characteristics [56]. It adapts the inertial weight based on the fitness value. The formula to calculate adaptive inertial weight is as follows:

$$\omega =\left\{ \begin{array}{l} \omega_{min}-(\omega_{max}-\omega_{min})*(f_{\mathrm{cur}}-f_{min})/(f_{avg}-f_{min}),f_{\mathrm{cur}}⩽f_{avg}\\ \omega_{max},f_{\mathrm{cur}}>f_{avg} \end{array}\right.$$ (13)

The present particle’s fitness value is given by f_cur_. The present population’s average fitness value is given by f_avg_. The lowest particle fitness value in the current population is given by f_min_.

### 4.3. Whale optimization algorithm

The WOA algorithm is a kind of bio-inspired meta-heuristic algorithm, proposed by Lewis and Mirjalili, that mimics the behavior of humpback whales [54]. Whales are exceptional creatures, not just because they are the largest mammals in the world but also because they are extremely intelligent and have developed social behaviors. The WOA algorithm employs a collection of randomly selected candidate solutions, and three rules are utilized to update and enhance the positions of these candidate solutions. The WOA has three steps: identification (encircling prey), exploitation (bubble-net attack), and exploration (search for prey), which will be discussed in the subsections below.

#### 4.3.1. Identification (encircling)

Humpback whales can detect their prey’s location and encircle it. Target hunting is assumed to be the best current candidate solution for the whale optimization algorithm because the optimal prey location in the search space is unclear. The reminder agents adjust their positions depending on the position of the best search prey, as illustrated below:

$$\vec{D}=|\vec{C}{\vec{X}}^{*}(t)-\vec{X}(t)|$$ (14)

$$\vec{X}(t+1)={\vec{X}}^{*}(t)-\vec{A}\vec{D}$$ (15)

where t represents the current iteration number, X* represents the best solution location vector, X represents the position vector, $\vec{X}(t+1)$ is the position of the whale at each iteration, and C and A are the coefficient vectors. Eqs (16) and (17) are calculated using coefficient vectors C and A.


$$\vec{A}=2\vec{a}\vec{r}-\vec{a}$$ (16)

$$\vec{C}=2\vec{r}$$ (17)

In the aforementioned equations, r represents a random vector with values between zero and one, and the value of a parameter is lowered linearly from two to zero during the exploitation and exploration steps.

#### 4.3.2. Exploitation (bubble-net attack)

Two mathematical models exist for humpback whale bubble-net behavior:

- The shrinking encircling mechanism: To activate the shrinking encircling mechanism, we must lower the value of a in Eq (16). It is important to note that the parameter a has a significant impact on the change in the range of A. During iterations, a parameter declines between zero and two, and A is a random number between -a and a. By picking random values for A between one and negative one, the updated search agent position can be anywhere among the primary agent positions and the best agent positions.
- The spiral updating position: This technique is used to calculate the distance between the X* and Y* coordinates of the prey and the X and Y coordinates of the whale. To imitate a humpback whale’s helical motion, spiral equations are expressed for the prey and whale’s positions:

$$\vec{D^{\prime }}=|\vec{X^{*}}(t)-\vec{X}(t)|$$ (18)

$$\vec{X}(t+1)=\vec{D^{\prime }}\cdot e^{bl}\cdot \cos\left(2\pi l\right)+\vec{X^{*}}(t)$$ (19)

where the constant b represents the spiral-shaped route path and L represents a number between negative one and one, which is chosen at random. Depending on Eq (20), the WOA chooses either the spiral-shaped or the encirclement mechanism to move the whales and each mechanism has a probability of 50%. The P parameter is assigned a random number between one and zero.


$$\vec{X}(t+1)=\left\{ \begin{array}{l} \vec{X^{*}}(t)-\vec{A}\cdot \vec{D}\;if\;p<0.5\\ \vec{D^{\prime }}\cdot e^{bi}\cdot cos\;(2\pi l)+{\vec{X}}^{*}(t)\;if\;p\geq 0.5 \end{array}\right.$$ (20)

#### 4.3.3. Exploration (search for prey)

The search behavior of humpback whales is random and is determined by the location of each individual whale relative to its neighbors. Therefore, vector A is employed with random values that are either more than one or less than one. The random movement of the whales is determined using Eqs (21) and (22).


$$\vec{D}=|\vec{C}\cdot \vec{X_{\mathrm{rand}}}-\vec{X}|$$ (21)

$$\vec{X}(t+1)=\vec{X_{\mathrm{rand}}}-\vec{A}\cdot \vec{D}$$ (22)

In the above equation, X_rand_ represents a random population position vector. A collection of random solutions is utilized to initiate the WOA algorithm. The positions of the search agents are modified depending on the best current solution or the random selection of the search agent in each iteration. Parameter a is reduced from two to zero to enable recognition of the exploitation and exploration phases. If $\vec{A}$ is less than or equal to one, the search agent is chosen at random. However, if $\vec{A}$ is more than one, the best solution is selected when the search agent’s position is modified. Based on the value of p, the algorithm chooses a circular or spiral motion.

### 4.4. Hybrid APSO-WOA algorithm

A hybrid APSO–WOA set is made up of independent APSO and WOA algorithms. WOA is employed for the exploration phase since its logarithmic spiral function covers more of an unclear search space. The exploration phase implies that an algorithm may attempt to provide several solutions. To solve a complicated non-linear issue, the particle position is changed to the whale position, which is identical to the particle position but is much more effective in moving the solution towards the optimal one. WOA accelerates the movement of particles toward the best value and decreases the computing time. As is known, APSO exploits the optimal solution in an unknown search space. Thus, obtaining the best feasible optimal solution to the problem, while avoiding local optima or local stagnation, is guaranteed by combining the best characteristics (exploitation with APSO and exploration with WOA). Moving towards the most optimal solutions, hybrid APSO–WOA combines the strengths of both WOA during the exploration phase and APSO during the exploitation phase.


$$v_{id}^{t+1}=\omega v_{id}^{t}+c_{1}r_{1}({\mathrm{Whale}\_\mathrm{Pos}}_{id}-x_{id}^{t})+c_{2}r_{2}({\mathrm{gbest}}_{d}-x_{id}^{t})$$ (23)

### 4.5. APSO-WOA-CNN and APSO-CNN algorithms

In this study, the APSO–WOA–CNN and APSO–WOA algorithms are utilized by the parameters of a 1D convolutional neural network and identify the right hyperparameters to minimize the high effort of manually tweaking the parameters that are needed to discover the detection task that is suited to the various types of IoT network attacks. The position of the particles is used to build each layer’s parameters in the CNN. Furthermore, the position parameter components for each dimension are set. Tables 2 and 3 illustrate the particle swarm parameter setting range.

**Table 2:** The value ranges of position parameters.

Particle Position	Hyperparameters	Description	Particle Value Interval	Data Type
$x_{i1}^{t}$	Con1-filter	Number of convolutional kernels	[100, 600]	Integer
$x_{i2}^{t}$	Con1-length	Length of convolutional filter	[1, 5]	Integer
$x_{i3}^{t}$	Con1-activation	Activation functions in the convolutional layer	relu(2), tanh(1), sigmoid(0)	Integer
$x_{i4}^{t}$	Con1-F2-dropout	Probability of nodes used between the convolutional and second layers	[0.4, 0.8]	Float
$x_{i5}^{t}$	F2-neuron	Number of second-layer neurons	[256, 1024]	Integer
$x_{i6}^{t}$	F2-activation	Activation functions in the second layer	relu(2), tanh(1), sigmoid(0)	Integer
$x_{i7}^{t}$	F3-neuron	Number of third-layer neurons	[256, 1024]	Integer
$x_{i8}^{t}$	F3-activation	Activation functions in the third layer	relu(2), tanh(1), sigmoid(0)	Integer
$x_{i9}^{t}$	Batch size	Batch sample size	[16, 200]	Integer
$x_{i10}^{t}$	Learning rate	Process of updating weights in the opposite direction	[0.01, 1]	Float

**Table 3:** The value ranges of velocity parameters.

Particle Velocity	Initial Value for Particle Interval	Data Type
$v_{i1}^{t}$	[1, 20]	Integer
$v_{i2}^{t}$	[0, 1]	Float
$v_{i3}^{t}$	[0, 1]	Float
$v_{i4}^{t}$	[0, 1]	Float
$v_{i5}^{t}$	[1, 100]	Integer
$v_{i6}^{t}$	[0, 1]	Float
$v_{i7}^{t}$	[1, 100]	Integer
$v_{i8}^{t}$	[0, 1]	Float
$v_{i9}^{t}$	[1, 20]	Integer
$v_{i10}^{t}$	[0, 1]	Float

**Fig 2:**
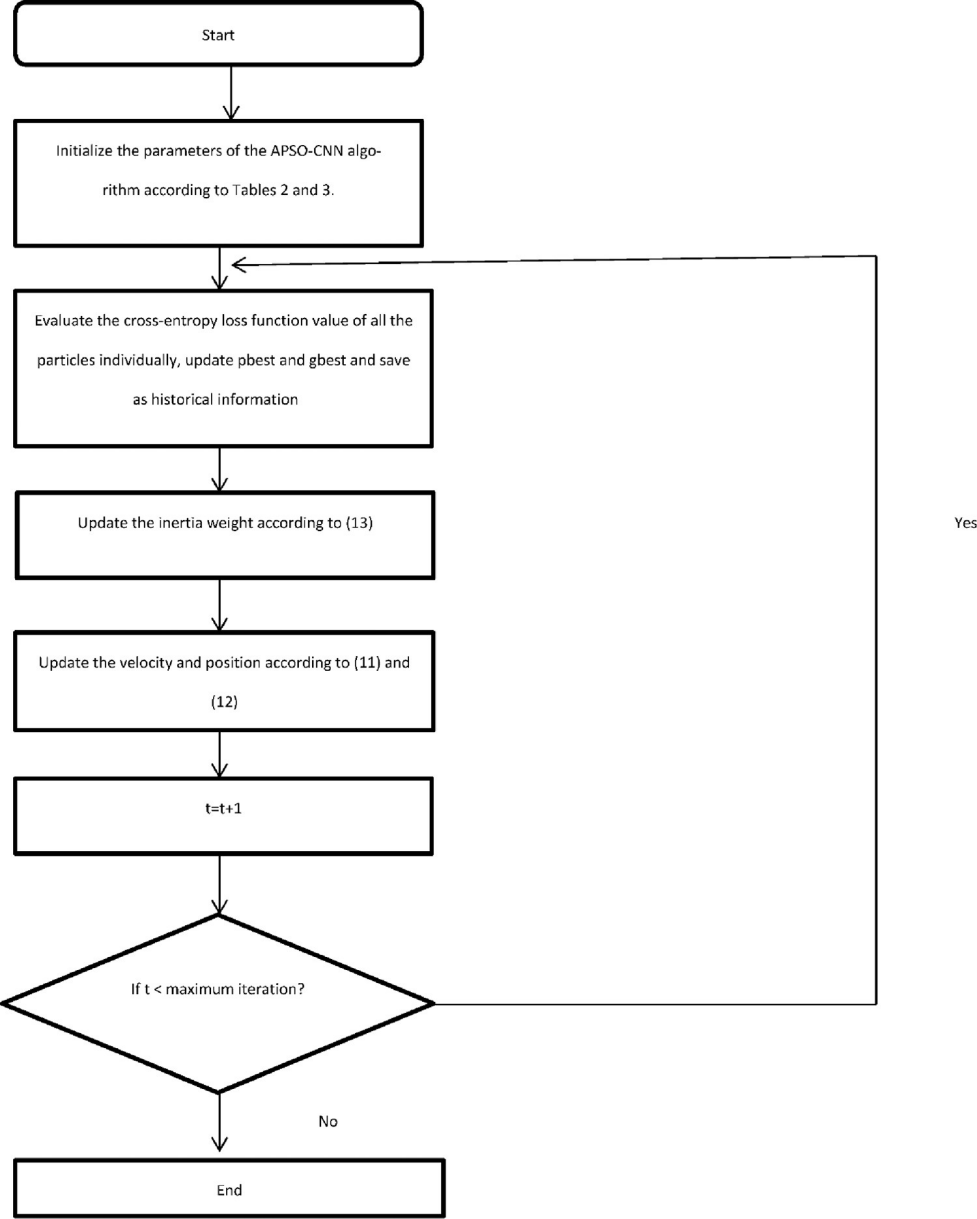
The APSO-CNN algorithm flowchart.

**Fig 3:**
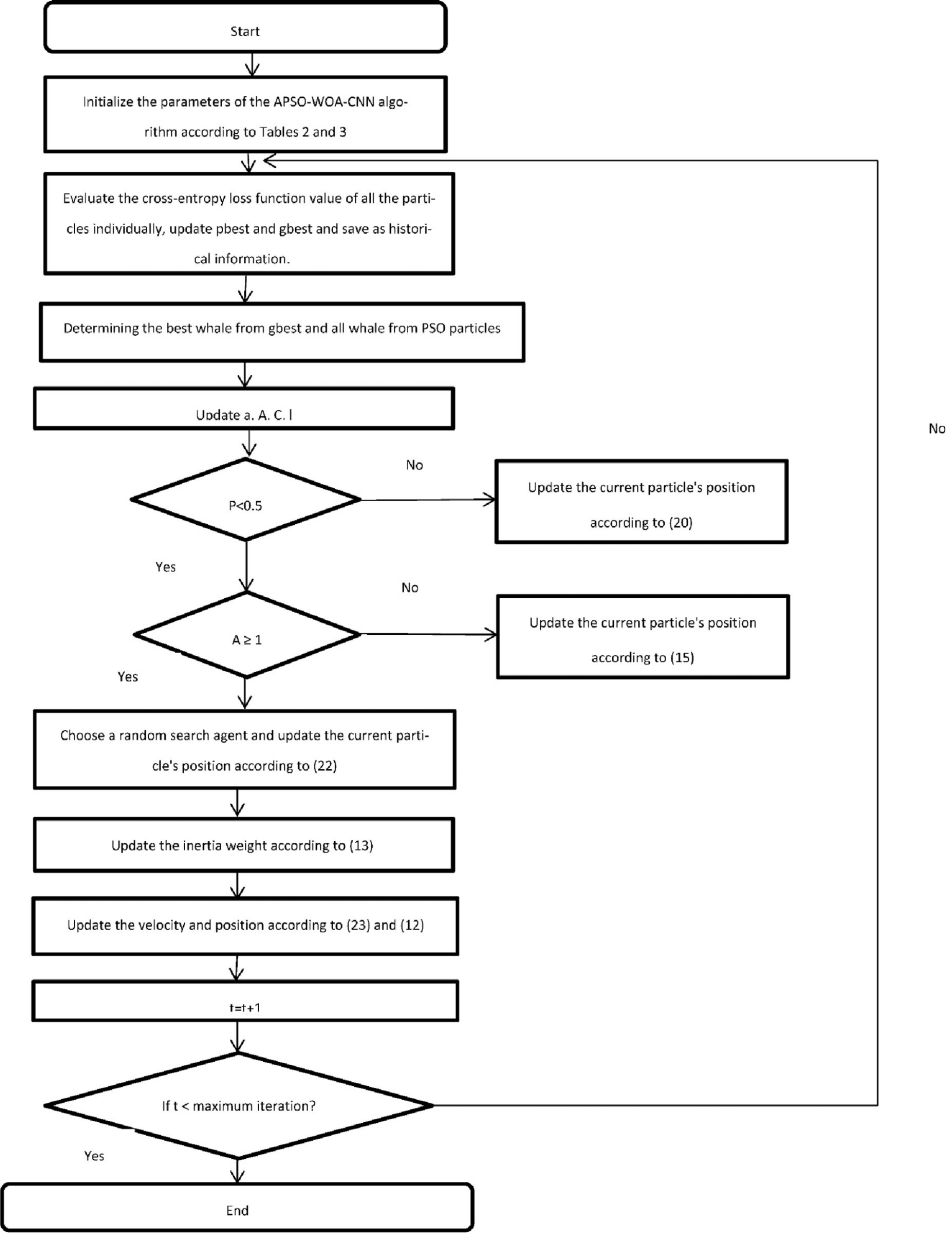
The APSO-WOA-CNN algorithm flowchart.

In Algorithm 1, we describe the procedure of the APSO–WOA–CNN algorithm to discover the best hyperparameters. In Algorithm 2, we describe the procedure of the APSO–CNN algorithm to discover the best hyperparameters. The position parameter of each particle correlates with the CNN network structure, and continuous iterative searches may be used to identify the best network structure parameters. In order to recognize all of the potential forms of IoT network attacks, researchers need to develop a model that has a powerful predictive analysis capacity. The loss function measures the variance between the actual and expected values. When the variance degree is at its lowest, the model performs well. The value of the cross-entropy function from the first training cycle of CNN is used as the fitness function value to evaluate particle quality and to reduce it. The softmax layer is used to determine each category’s prediction probability. Fig 2 illustrates the APSO–CNN flowchart. Fig 3 illustrates the APSO–WOA–CNN flowchart.

Algorithm 1. The APSO-WOA-CNN Algorithm to Determine the Hyperparameters.

*Input: N = Total populations, d,* *t*_max_

1. Generate the position parameter $x_{id}^{t}$ depending on Table 2.

2. Generate the velocity parameter $v_{id}^{t}$ depending on Table 3.

3. **While** (t < t_max_**)**

4.    Calculate the fitness for each particle, identify *f*_*min*_, *f*_*avg*_, *f*_cur_.

5.    Update gbest_*d*_, and pbest_*id*_.

6.    X* = gbest_*d*_.

7.    **For** each particle in APSO particles.

8.        *Update a*, *A(*Eq (16)), *C (*Eq (17)), *l*.

9.        ***If 1****(p < 0*.*5)*.

10.            ***If2*** *(*|*A*|< 1*)*.

11.                Update the current particle’s position by (Eq (15)).

12.            ***Else if2*** *(*|*A*| ≥ 1*)*.

13.                Choose a random search agent $\vec{X_{\mathrm{rand}}}$.

14.            Update the current particle’s position by (Eq (22)).

15.            ***End if2***.

16.        ***Else if1*** (*p* ≥ 0.5)

17.        Update the current particle’s position by (Eq (20)).

18.        ***End if1***.

19.        ***End for***.

20.        Update the value of W depending on (Eq (13)).

21.        Update the velocity $v_{id}^{t+1}$ and position $x_{id}^{t+1}$ depending on (Eq (23)) and (Eq (12)).

23.        t = t + 1.

24.    **End while**.

25.    **Return** X*.

Algorithm 2. The APSO-CNN Algorithm to Determine the Hyperparameters.

*Input*: *N* = *Total populations, d,* *t*_max_

1. Generate the position parameter $x_{id}^{t}$ depending on Table 2.

2. Generate the velocity parameter $v_{id}^{t}$ depending on Table 3.

3. **While** (t < t_max_)

4.        Calculate the fitness for each particle, identify f_min_, f_avg_, f_cur_.

5.        Update pbest_id_, and gbest_d_.

6.        Update the value of W depending on (Eq (13)).

7.        Update the velocity $v_{id}^{t+1}$ and position $x_{id}^{t+1}$ depending on (Eqs (11) and (12)).

9.        t = t + 1.

10. **End while**.

11. **Return** gbest_d_

As illustrated in Fig 2, the APSO-CNN algorithm’s position parameters are initialized in the first phase based on Tables 2 and 3. During the first training period, the value of the cross-entropy loss function is calculated using 10 position parameters for each particle to figure out the fitness value of each particle for the current iteration. Store each particle’s minimal fitness value, the global minimum, and compute the average. The inertia weight is changed in the third stage using the Eq (13). The fourth stage involves updating the position and velocity parameters in the APSO-CNN using the Eqs (11) and (12). Increase the number of iterations by one in the fifth step. The iteration should proceed to the next step if the extreme number of iterations has not been reached. If not, the process should end by stopping the loop and putting out the fitness value with the lowest score.

As illustrated in Fig 3, the APSO-WOA-CNN algorithm’s position parameters are initialized in the first phase based on Tables 2 and 3. During the first training period, the value of the cross-entropy loss function is calculated using 10 position parameters for each particle to figure out the fitness value of each particle for the current iteration. Store each particle’s minimal fitness value, the global minimum, and compute the average. The best whale from gbest and all whale from PSO particles are determined in the second step. In the third step, update all the values of a, A, C, and l. If the p value is less than 0.5, the current particle’s position is updated using Eqn (20). otherwise, If is less than or equal to one, the search agent is chosen at random using Eq (22). However, if there is more than one, the best solution is selected when the search agent’s position is modified using Eq (15). The inertia weight is changed in the next stage using Eq (13). The next stage involves updating the position and velocity parameters in the APSO-CNN using Eqs (11) and (12). In the next step, increase the number of iterations by one. The iteration should proceed to the next step if the extreme number of iterations has not been reached. If not, the process should end by stopping the loop and putting out the fitness value with the lowest score.

## 5. Experiments

### 5.1. Hardware and software

The Python 3.7 and Keras 2.3 libraries were used to carry out all of the experiments, and the models were trained on an NVIDIA Quadro P1000 Super GPU (4 GB), which had 32 GB of RAM and an eight-core Xeon E5-2690 processor.

### 5.2. Simulation and discussion

**Fig 4:**
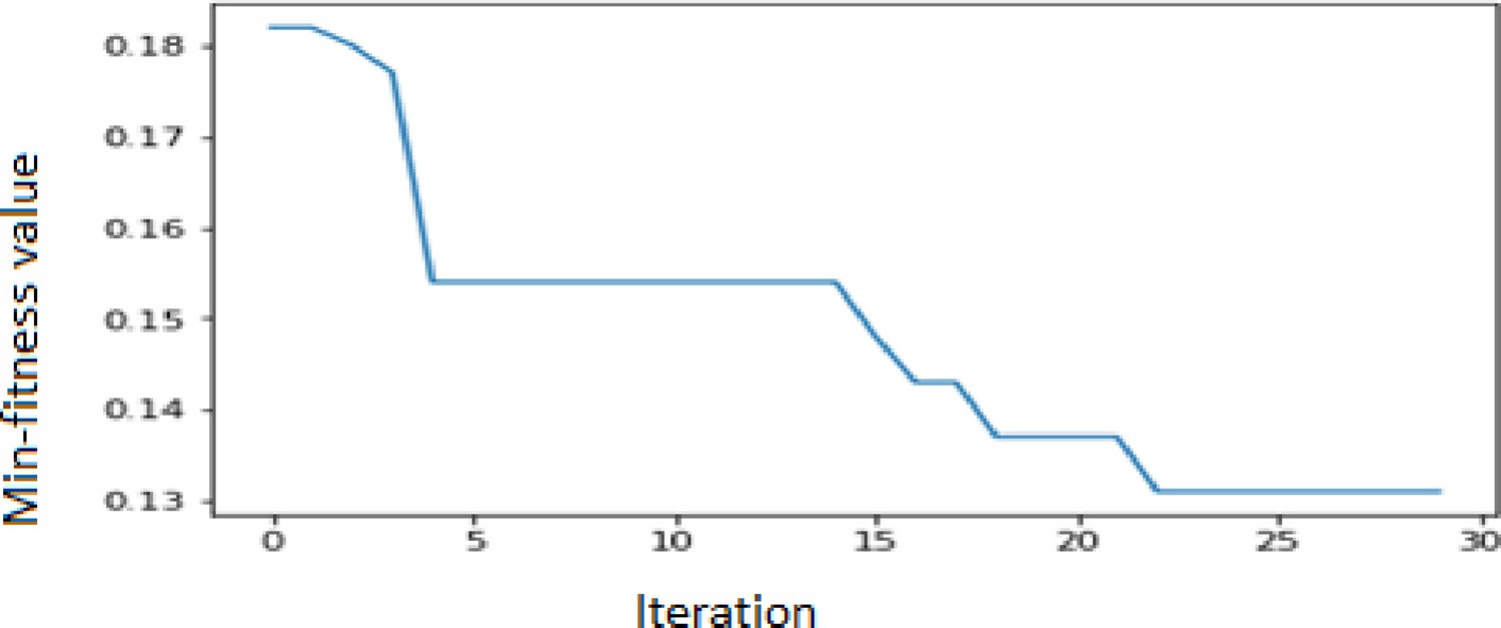
APSO–WOA–CNN algorithm’s minimal fitness values modified over iterations.

**Fig 5:**
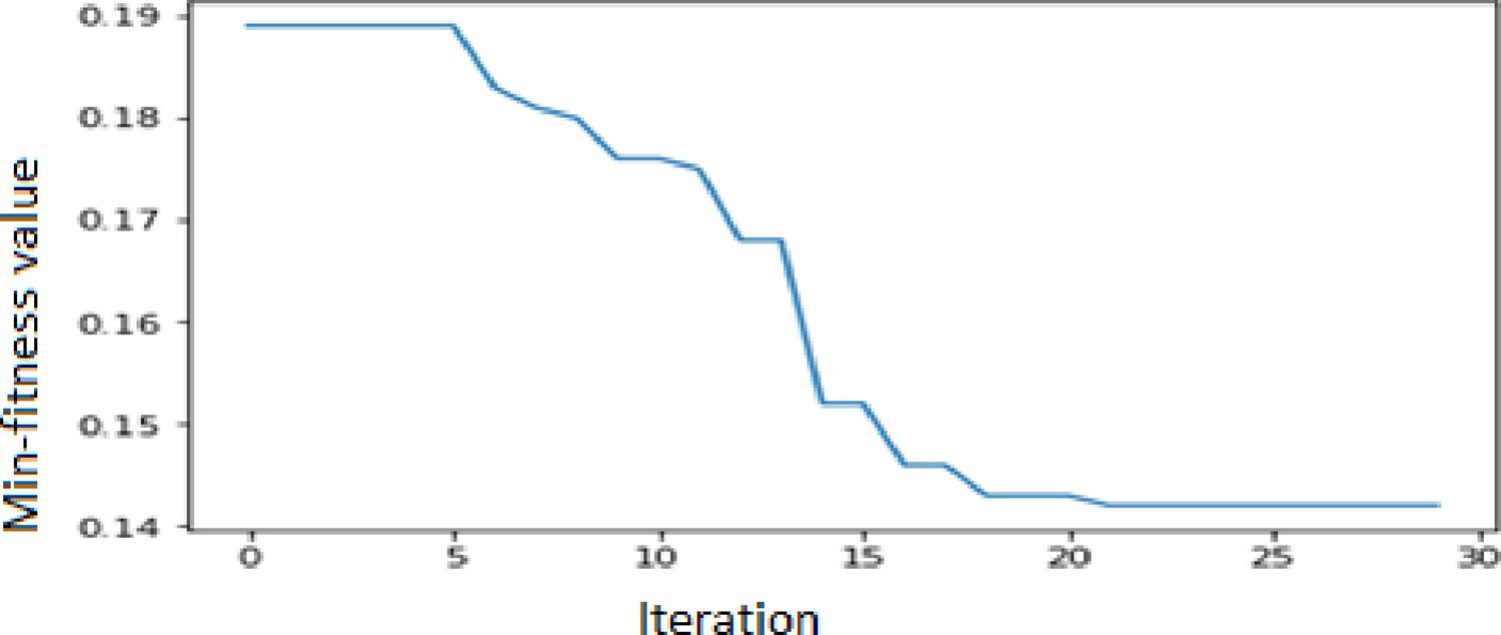
APSO–CNN algorithm’s minimal fitness values modified over iterations.

In this study, the APSO–WOA–CNN and APSO–CNN algorithms’ parameter settings were configured as follows. The size of the population was 20, the maximum number of particle swarm iterations was 30, the C1 and C2 coefficient values were both 2, the maximum weight of inertia was 0.9, and the minimum weight of inertia was 0.4. As illustrated in Fig 4, the APSO–WOA–CNN algorithm’s minimal fitness value was adjusted over iterations. As illustrated in Fig 5, the APSO–CNN algorithm’s minimal fitness value was adjusted over iterations.

The best particle fitness value was achieved by the APSO–CNN algorithm at 0.1431772 and by the APSO–WOA–CNN algorithm at 0.1319339 after iterative optimization. Thus, the APSO–WOA–CNN performed better than the APSO–CNN algorithm. Based on the best particle location, each dimension’s components may be identified. Table 4 shows the CNN’s optimal hyperparameters using the APSO–CNN algorithm. Table 5 shows the CNN’s optimal hyperparameters using the APSO–WOA–CNN algorithm.

**Table 4:** CNN’s optimal hyperparameters utilizing the APSO–CNN algorithm.

Particle Position	Optimization Value
X_1_	600
X_2_	5
X_3_	relu
X_4_	0.4
X_5_	256
X_6_	sigmoid
X_7_	295
X_8_	relu
X_9_	16
X_10_	0.1

**Table 5:** CNN’s optimal hyperparameters utilizing the APSO-WOA-CNN algorithm.

Particle Position	Optimization Value
x_1_	600
X_2_	1
X_3_	Relu
X_4_	0.4
X_5_	399
X_6_	tanh
X_7_	265
X_8_	Relu
X_9_	16
X_10_	0.07

Putting in the parameters given in Tables 4 and 5, which come from the APSO–WOA–CNN and APSO–CNN algorithms, into the network structure shown in Section 3.1 increases the value of the accuracy and loss function of the validation and training datasets throughout each training cycle for both the APSO–WOA–CNN and APSO–CNN algorithms. The benefits of the proposed method are also apparent in comparison to the same four indices generated from FNN training with manually chosen parameters. Additionally, we compared the CNN’s optimal hyperparameters using the APSO–CNN algorithm and the CNN’s optimal hyperparameters using the APSO–WOA–CNN algorithm. In order to expedite the training process, an early stop mechanism was established in the network structure program. The training was halted and the best model of the network was preserved if the validation set accuracy did not increase during one training cycle.

**Fig 6:**
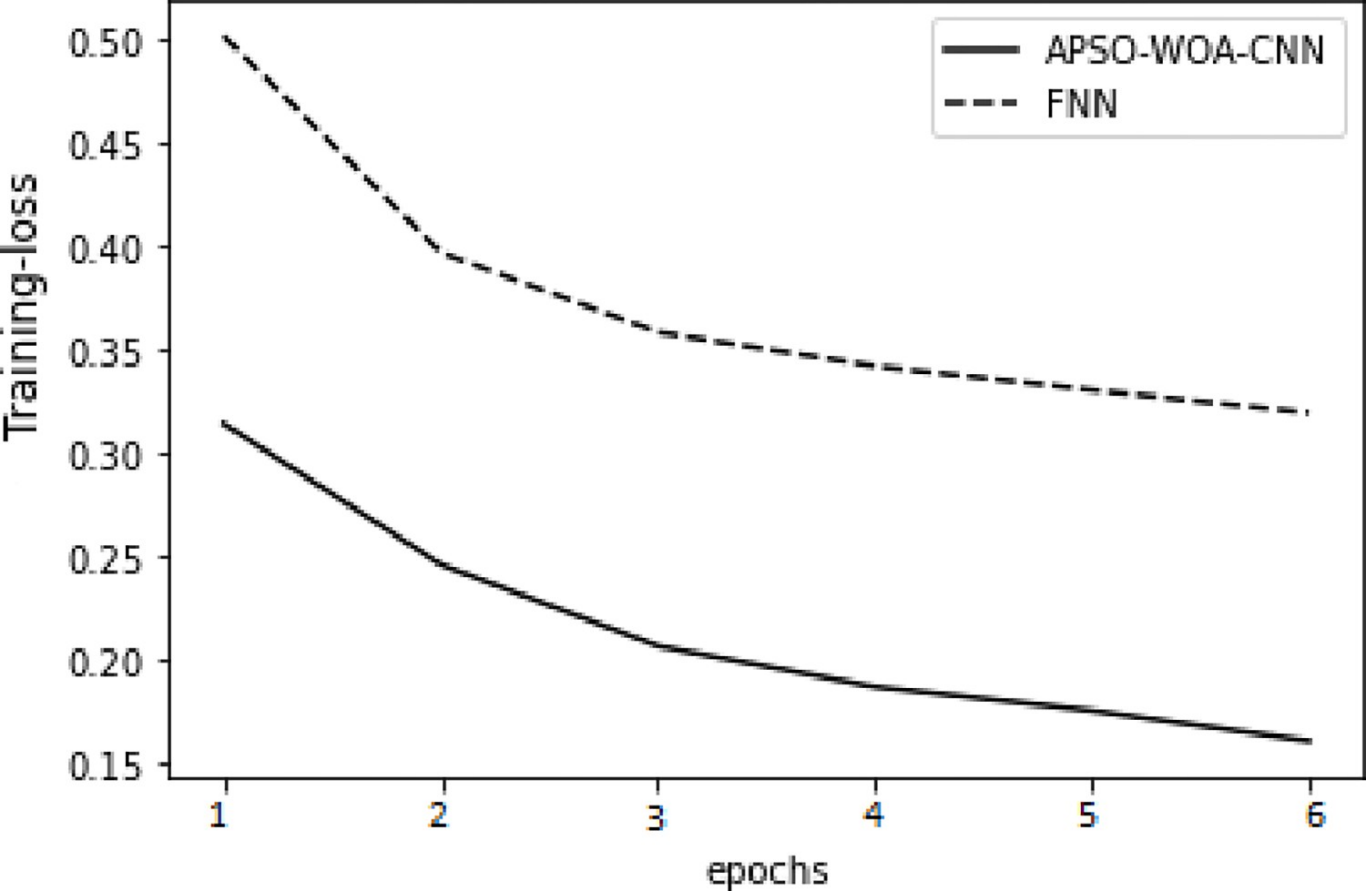
APSO–WOA–CNN and FNN algorithms’ loss function training sets.

**Fig 7:**
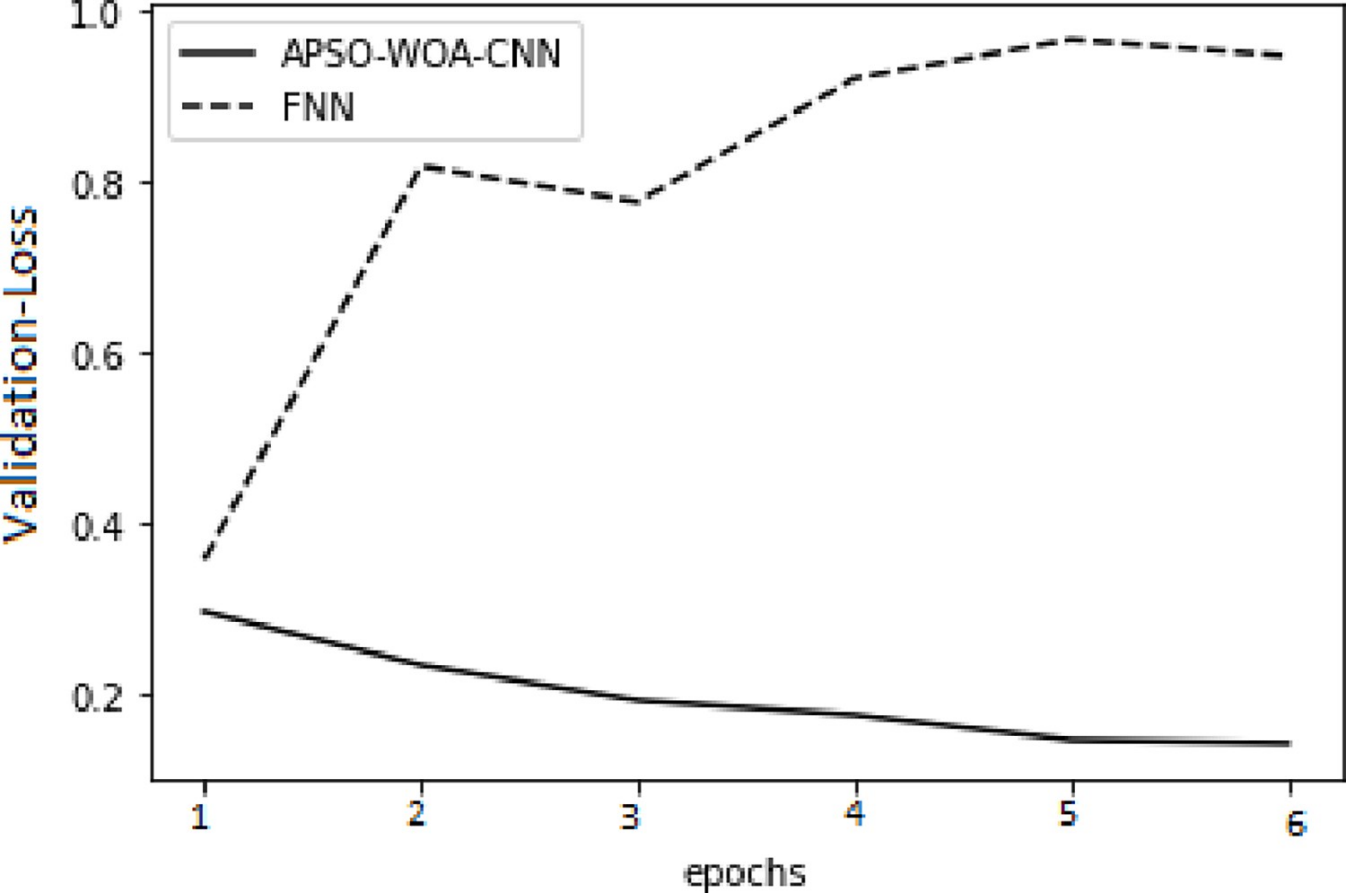
APSO–WOA–CNN and FNN algorithms’ loss function validation sets.

**Fig 8:**
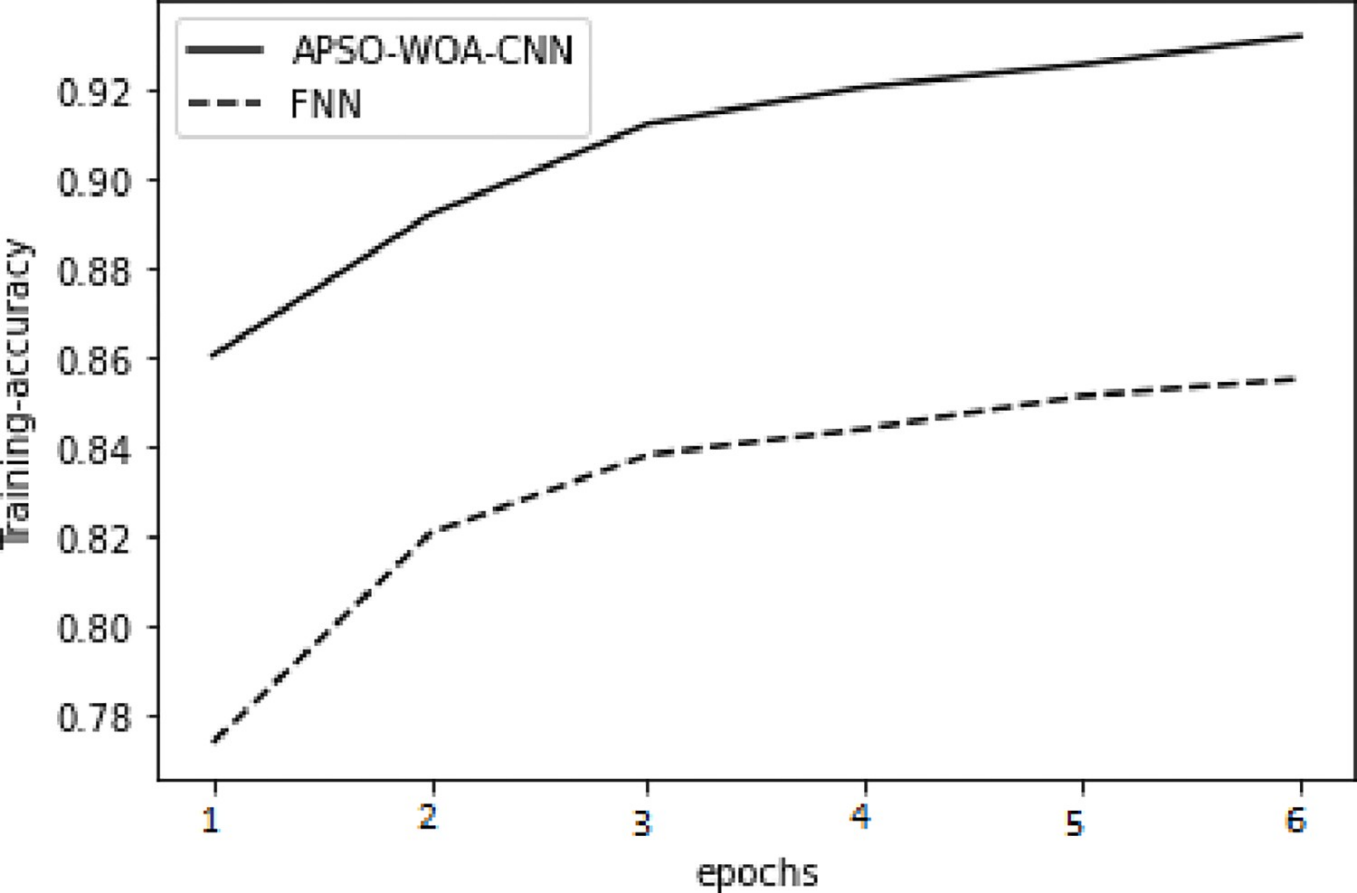
APSO–WOA–CNN and FNN algorithms’ accuracy training sets.

**Fig 9:**
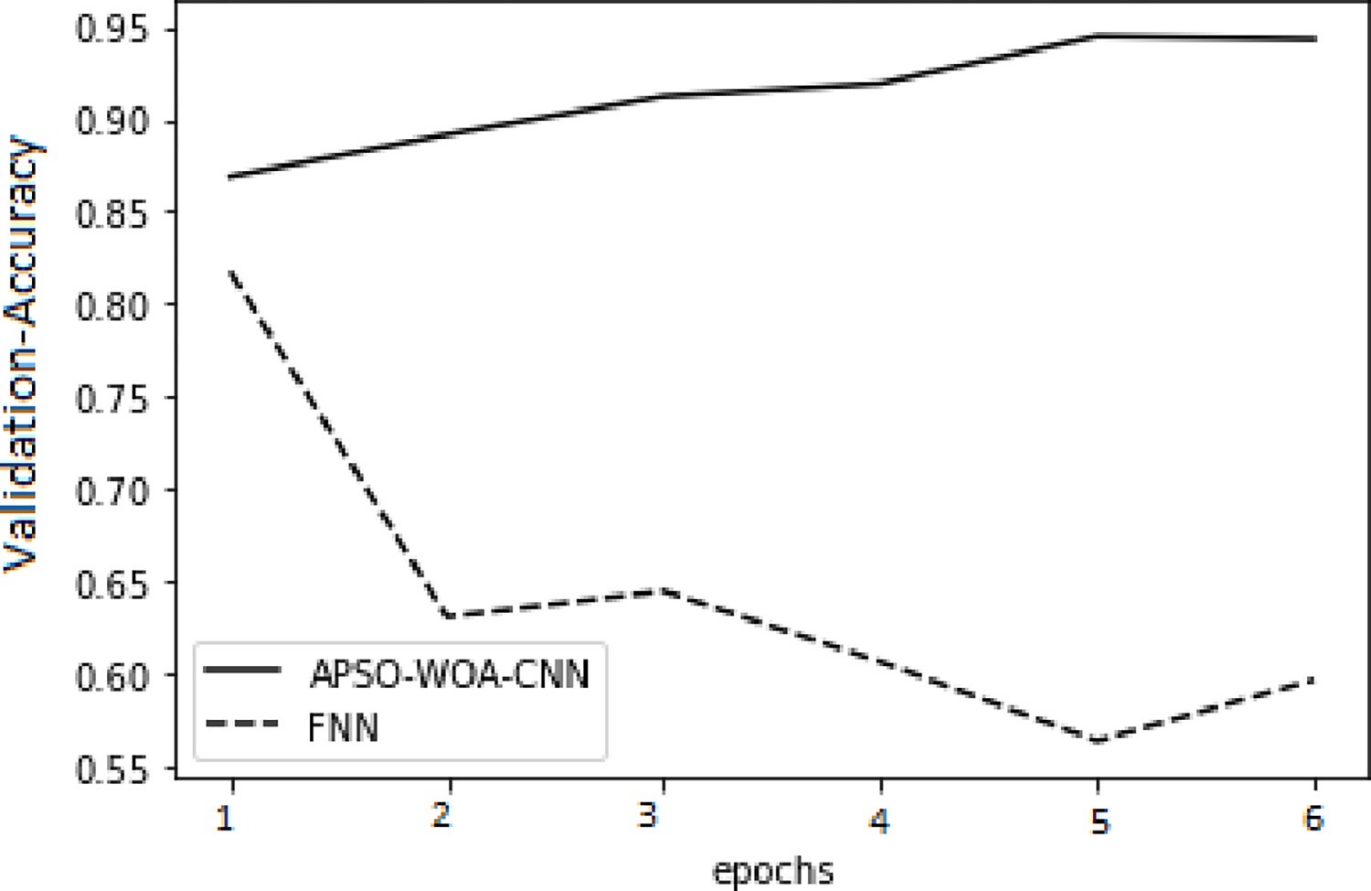
APSO–WOA–CNN and FNN algorithms’ accuracy validation sets.

As illustrated in Figs 6 and 7 in the training set, the value of the APSO–WOA–CNN algorithm’s cross-entropy loss was much lower than that of the FNN algorithm, but its accuracy was significantly greater. This shows that the proposed APSO–WOA–CNN algorithm had a better training effect. Similarly, as illustrated in Figs 8 and 9 in the validation set, the proposed APSO–WOA–CNN algorithm achieves higher accuracy and decreases the value of the cross-entropy loss on the validation set.

**Fig 10:**
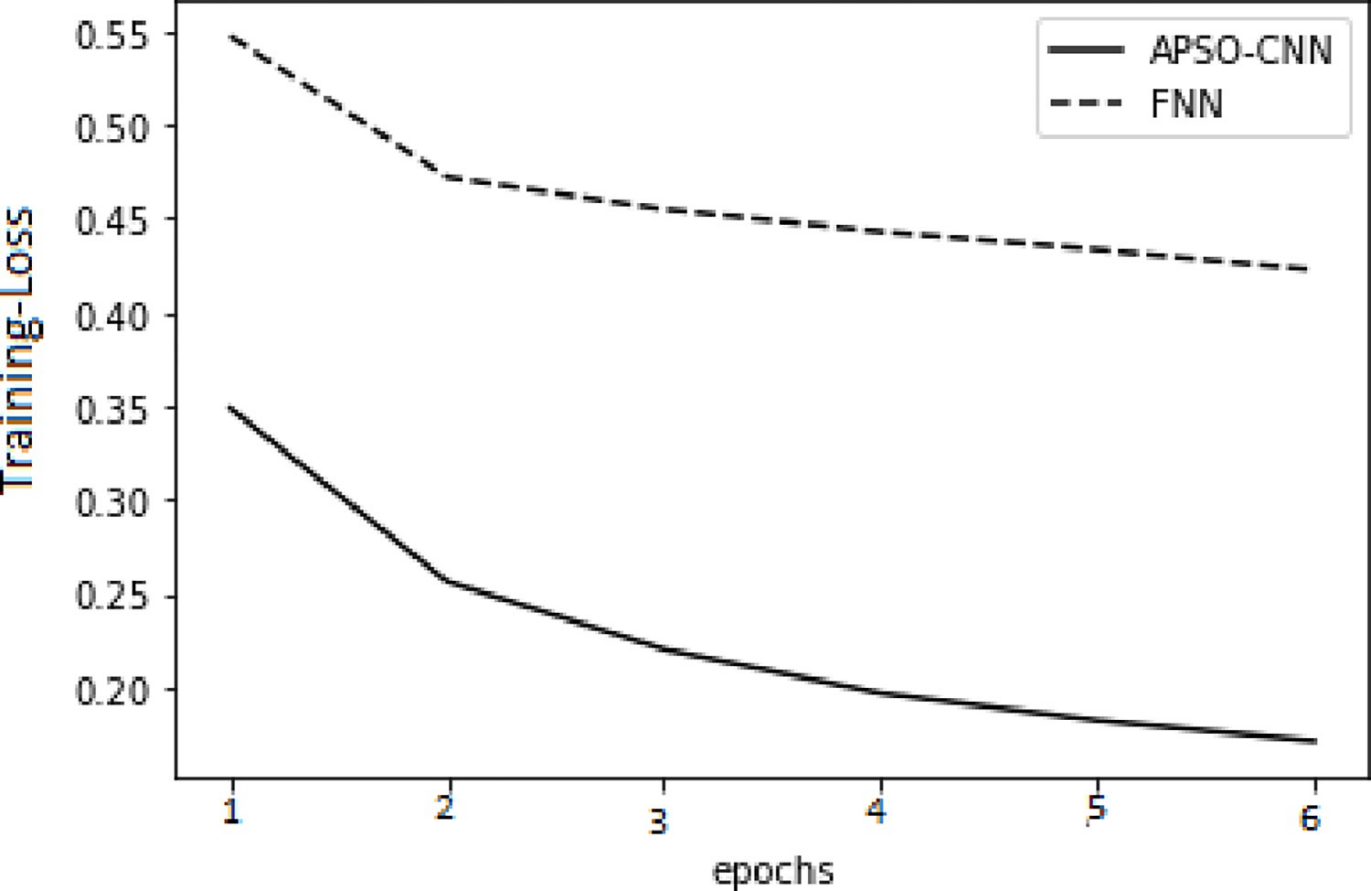
APSO–CNN and FNN algorithms’ loss function training sets.

**Fig 11:**
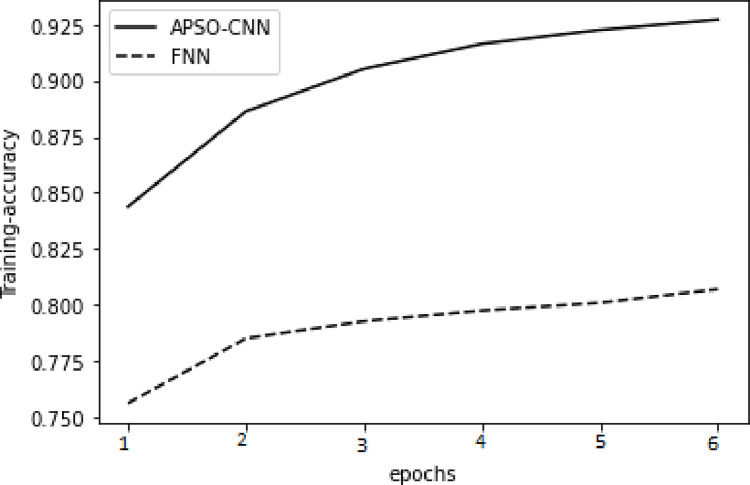
APSO–CNN and FNN algorithms’ accuracy training sets.

**Fig 12:**
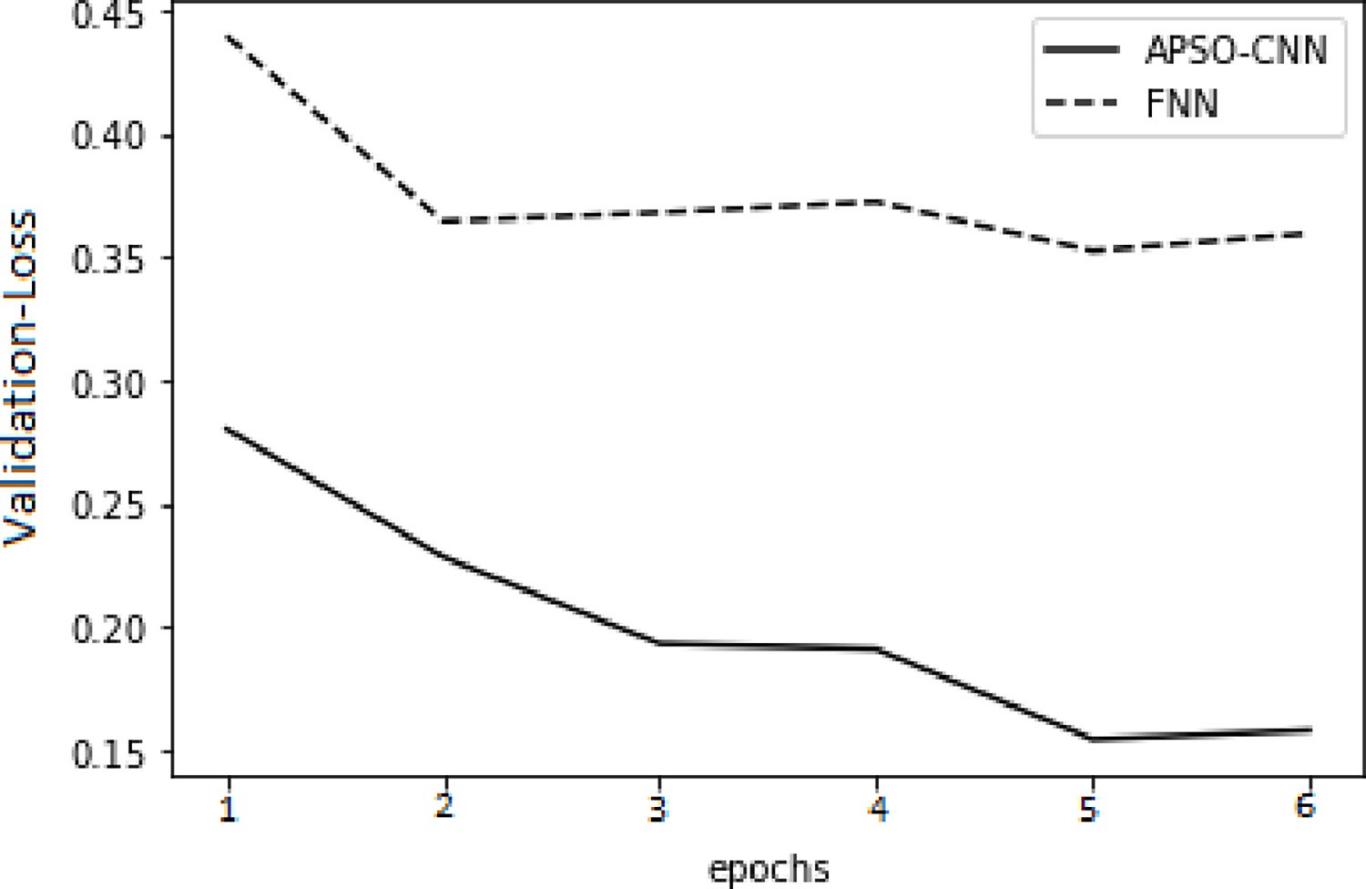
APSO–CNN and FNN algorithms’ loss function validation sets.

**Fig 13:**
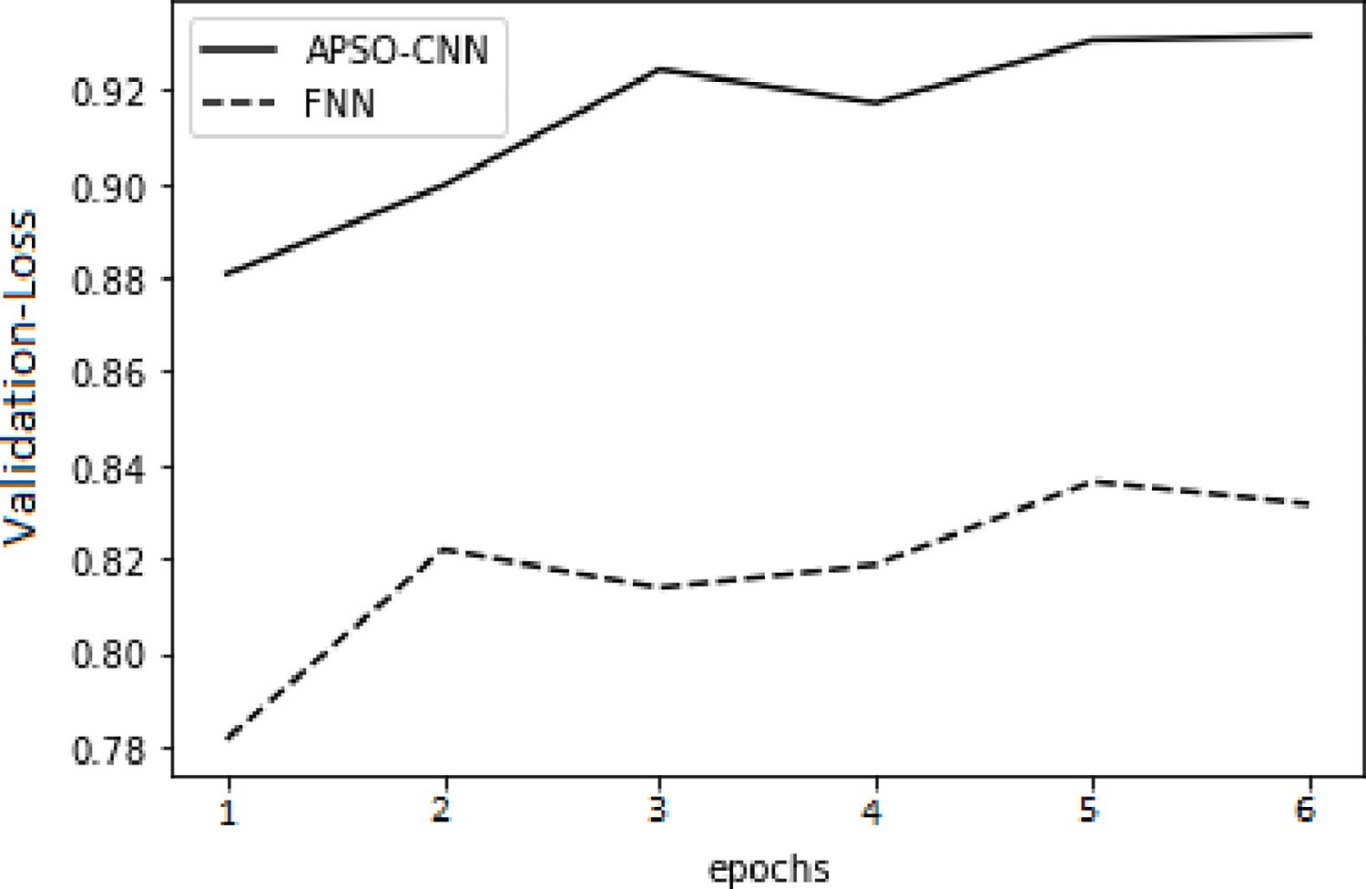
APSO–CNN and FNN algorithms’ accuracy validation sets.

In respect of the validation set and the training set, higher accuracy and lower cross-entropy loss were attained through the APSO-CNN algorithms compared to the FNN algorithm, as illustrated in Figs 10–13. This shows that the suggested APSO-CNN algorithm has a greater training impact, demonstrating its effectiveness in training models.

**Fig 14:**
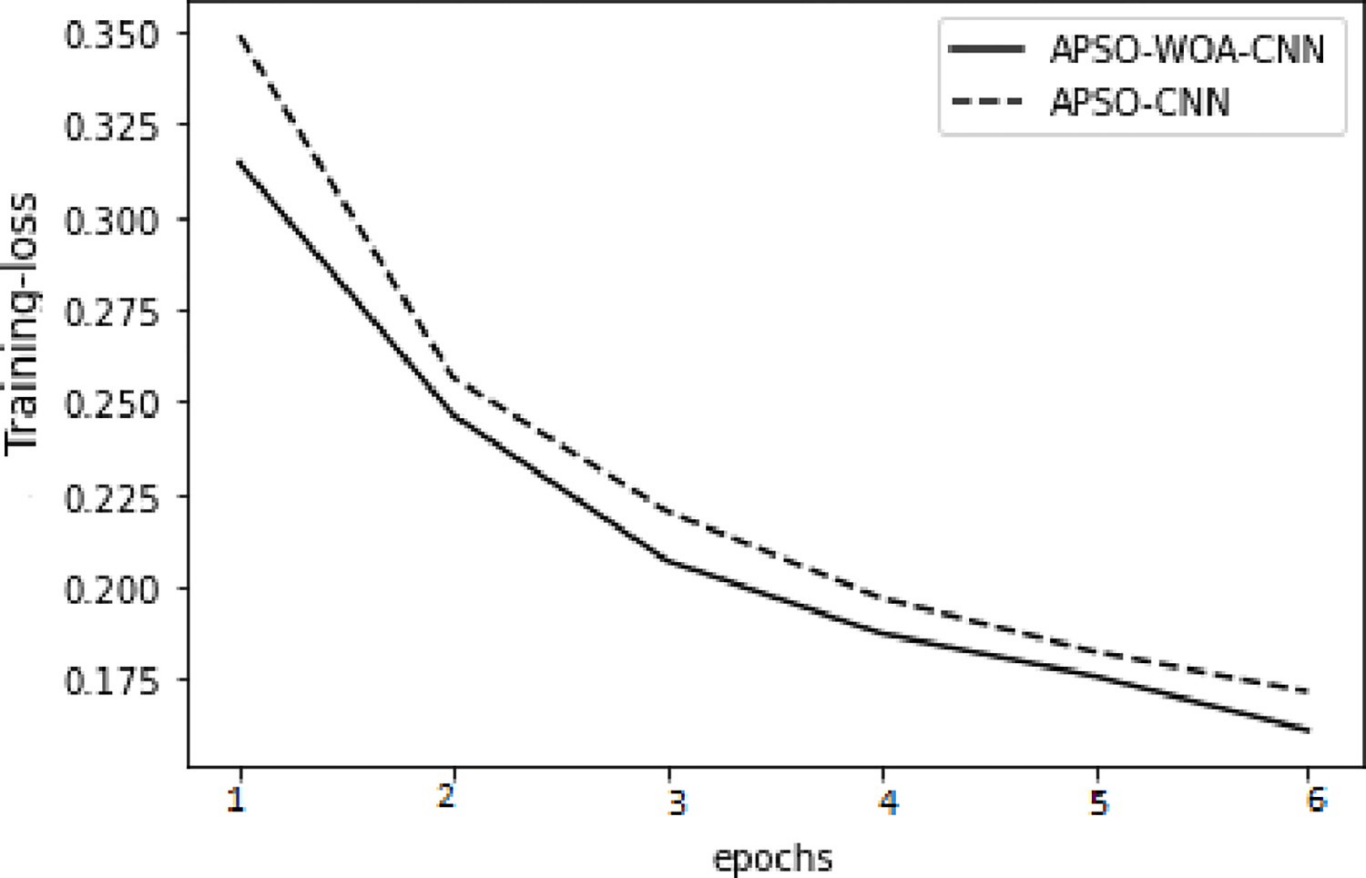
APSO–WOA–CNN and APSO–CNN algorithms’ loss function training sets.

**Fig 15:**
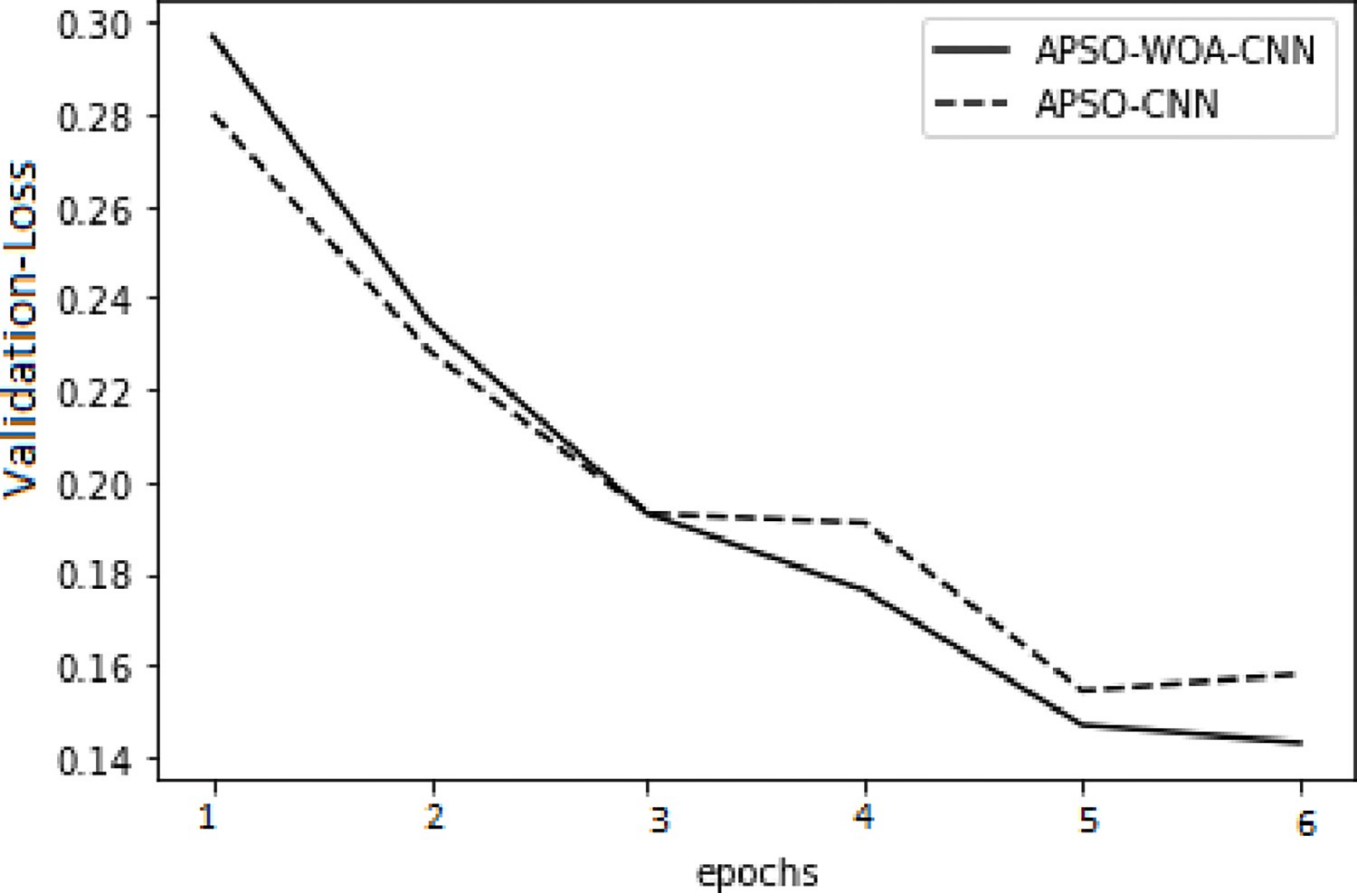
APSO–WOA–CNN and APSO–CNN algorithms’ loss function validation sets.

**Fig 16:**
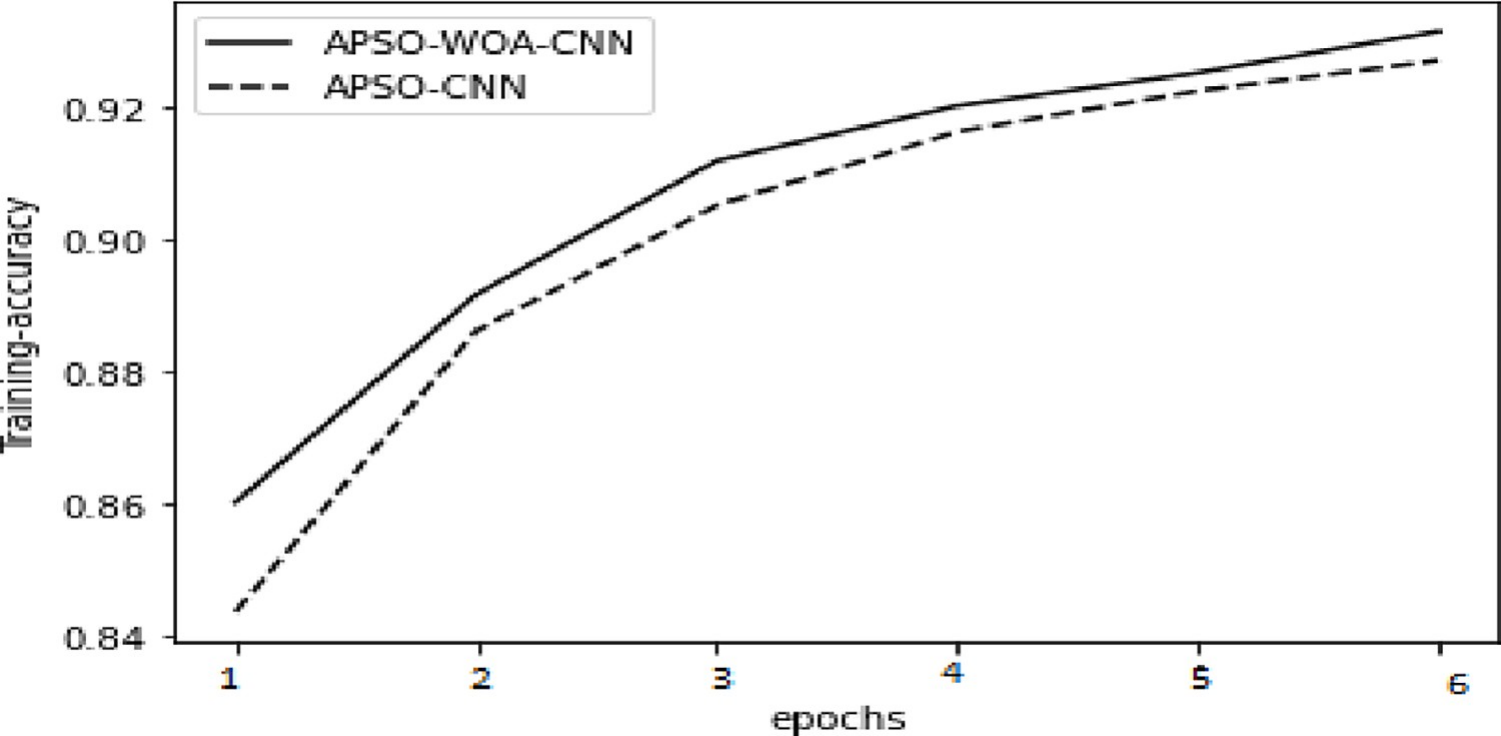
APSO–WOA–CNN and APSO–CNN algorithms’ accuracy training sets.

**Fig 17:**
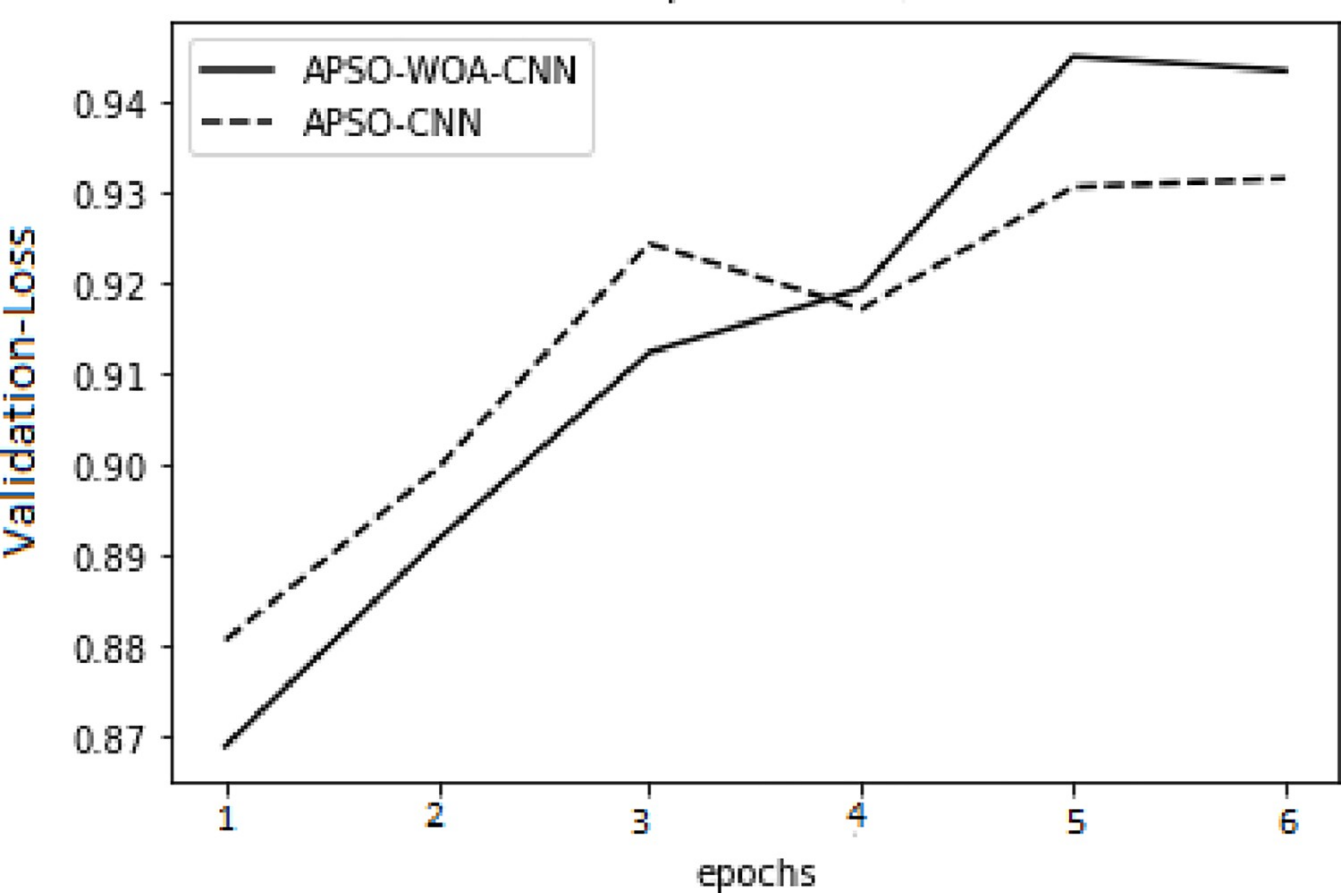
APSO–WOA–CNN and APSO–CNN algorithms’ accuracy validation sets.

In the model training, the APSO–WOA–CNN and APSO–CNN algorithms performed significantly better than the FNN algorithm with higher accuracy, and the models’ overall detection rates were also better. The APSO–WOA–CNN algorithm has higher accuracy and a smaller cross-entropy loss than the APSO-CNN algorithm, as illustrated in Figs 14–17. Thus, the APSO–WOA–CNN algorithm can detect multi-type network attacks better than APSO–CNN algorithms.

### 5.3. Results and performance analysis

To determine the successfulness of various classification approaches to identify network attacks, we evaluated both the APSO–WOA–CNN and APSO–CNN algorithms against SVM and FNN. Then, five evaluation indicators were used to evaluate the performance of four algorithms.

#### 5.3.1. Classification accuracy

$$\mathrm{accuracy}=n_{\mathrm{true}}/n_{\mathrm{total}}$$ (24)

where n_true_ represents the number of corrected samples classified, and the total sample is represented by n_total_.

Accuracy measures the fraction of properly classified samples among all samples. In addition, a high percentage of the correct numbers must be found, which means the model is effective and dependable for IoT attack detection.

#### 5.3.2. Kappa coefficient

$$kappa=(\mathrm{accuracy}-p_{e})/(1-p_{e})$$ (25)

where $\rho_{e}=(\sum_{i=1}^{\mathrm{dass}}\;n_{i}^{\mathrm{label}\_\mathrm{rure}}\times n_{i}^{\mathrm{predict}\_\mathrm{true}})/(n_{\mathrm{total}}\times n_{\mathrm{total}})$ represents the actual quantum of samples, and $n_{i}^{\mathrm{label}\_\mathrm{rure}}$represents the predicted quantum of samples.

The kappa coefficient evaluates whether the model’s prediction outcomes and actual classification results are consistent. The values might be between negative one and one. However, in practice, the values might be between zero and one. The kappa coefficient’s accuracy can only be used to detect the correct overall network mode. Obviously, the greater the value of the kappa coefficient, the greater the consistency achieved and the more effective the model’s classification impact.

#### 5.3.3. Average precision

$$\mathrm{ave}-\mathrm{precision}=(1/\mathrm{class})\times [\sum_{i=1}^{\mathrm{class}}\;n_{i}^{\mathrm{true}}/(n_{i}^{\mathrm{true}}+n_{i}^{\mathrm{false}})]$$ (26)

where class indicates the different types of network attacks, $n_{i}^{\mathrm{true}}$ is the division in the number of corrected samples and the i-th category, and $n_{i}^{\mathrm{false}}$is the division of the number of error samples and i-th category.

The number of network traffic types found in each actual network traffic class is the average precision. For this study, it was necessary to detect particular network attack types, which can dictate subsequent steps. It was also necessary to determine the accuracy of the detection for each different network attack type. Due to this, the low average detection accuracy across all the categories illustrates the high identification rate of each network attack type.

#### 5.3.4. Hamming loss

$$\mathrm{hamming}-\mathrm{loss}=(1/n_{\mathrm{total}})\times [\sum_{i=1}^{n_{\mathrm{total}}}\;count(y_{i}^{\mathrm{true}}⊕y_{i}^{\mathrm{predict}})/\mathrm{class}]$$ (27)

where n_total_ is the number of total samples, $y_{i}^{\mathrm{true}}$ is the i-th sample’s actual label, count(.) indicates the number of one, class indicates the quantum of sample categories, and $y_{i}^{\mathrm{predict}}$ is the i-th predicted label.

Multiple classifications are a challenge that can be solved using Hamming loss. This calculates the distance between the actual and predicted label. Its values are in the range [0,1]. A value equal to one denotes that the model has the opposite outcome to what is expected, and a value equal to zero denotes that the predicted and real results are the same. For practical purposes, the model was solved to accurately predict the type of network attack. Hamming loss is the count of actual labels that do not match the predicted labels. In addition, Hamming loss may validate the model’s ability to accurately identify real network attack types. As a result, with respect to detecting network attacks, a classifier is considered to be more effective when its indication is less significant.

#### 5.3.5. Jaccard similarity coefficient

$$J=(1/n_{\mathrm{total}})\times \left(\sum_{i=1}^{n_{\mathrm{total}}}\;|y_{i}^{\mathrm{true}}\cap y_{i}^{\mathrm{predict}}|/|y_{i}^{\mathrm{true}}\cup y_{i}^{\mathrm{predict}}|\right)$$ (28)

From the formula’s characteristics, it can be observed that when the predicted label matches the actual label, the value is one or zero. Therefore, the Jaccard similarity coefficient reflects the model’s quality.

**Fig 18:**
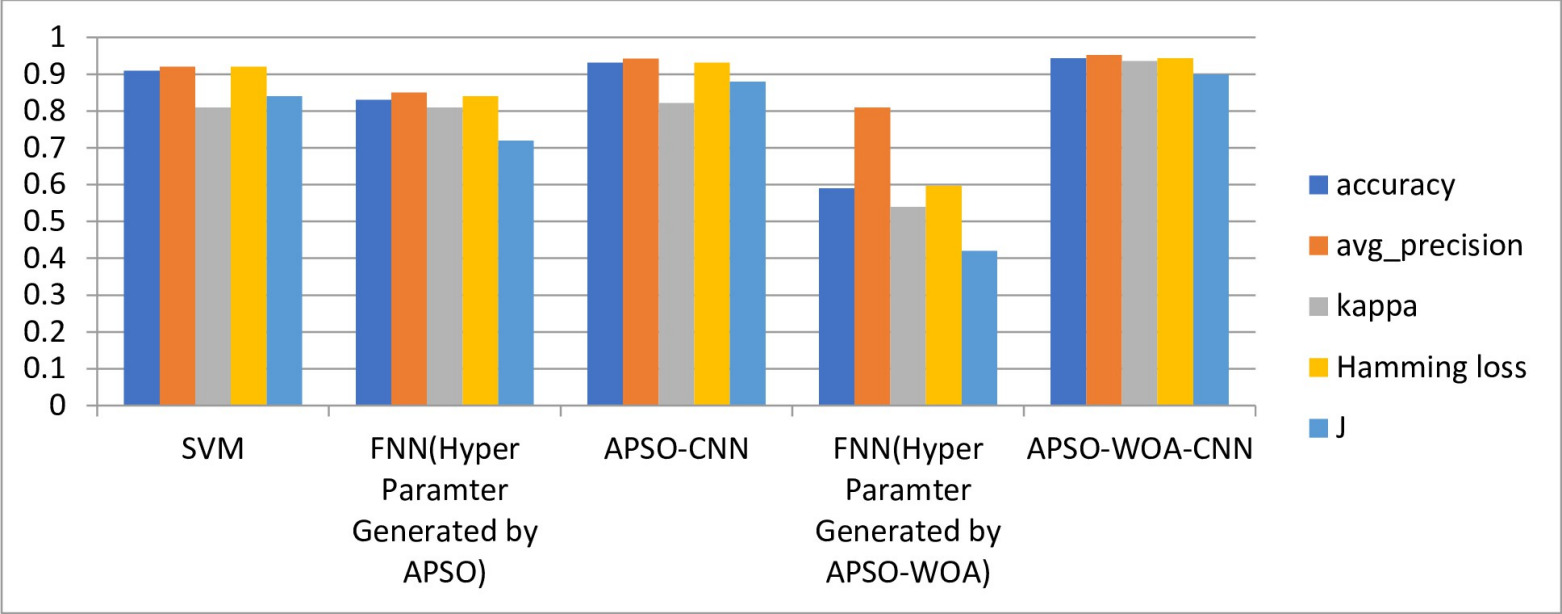
Evaluation index performance among algorithms.

The proposed APSO–WOA–CNN and APSO–CNN algorithms were compared to SVM and FNN using the five evaluation metrics listed above. The results of the comparison using five indicators for each algorithm are shown graphically in Fig 18. The results show that the APSO–WOA–CNN algorithm improves accuracy by 1.25%, average precision by 1%, the kappa coefficient by 11%, Hamming loss by 1.2%, and the Jaccard similarity coefficient by 2%. Compared to the APSO-CNN algorithm, the APSO-CNN algorithm has the highest performance of the algorithms. Thus, the APSO–WOA–CNN algorithm can detect multi-type network attacks better than other algorithms. Tables 6 illustrate the results of several measures of SVM, FNN, APSO-CNN, and APSO-WOA-CNN. Table 7 compares the results of the proposed model with other methods using the same datasets. Tables 6 and 7 show that the proposed model, APSO-WOA-CNN, is more accurate than other methods.

**Table 6:** Results of all algorithms according to evaluation matrices.

Methods	accuracy	avg_precision	kappa	Hamming loss	J
SVM	0.91	0.92	0.81	0.92	0.84
FNN(Hyper Paramter Generated by APSO)	0.83	0.85	0.81	0.84	0.72
APSO-CNN	0.931	0.942	0.822	0.932	0.88
FNN(Hyper Paramter Generated by APSO-WOA)	0.59	0.81	0.54	0.597	0.42
APSO-WOA-CNN	0.9454	0.952	0.936	0.944	0.9

**Table 7:** Comparison of proposed model with other methods using same datasets.

Methods	Accuracy %
Isolation Forest [75]	88.32
ANN [76]	92
LGBA-NN [70]	90
BA-NN [70]	85.5
PSO-NN [70]	85.2
LSTM [77]	94.2
SVM	91
APSO-WOA-CNN	94.5

## 6. Discussion

In this study, we proposed a novel hybrid meta-heuristic APSO-WOA for optimization of the hyperparameters of a CNN (APSO-WOA-CNN). With the proposed APSO-WOA optimization algorithm, the hyperparameters of a 1D CNN structural network can be optimized. The APSO–WOA optimization algorithm’s fitness value is defined as the validation set’s cross-entropy loss function during CNN model training. We compare our proposed optimization algorithm with others, such as the APSO algorithm, for optimization of the hyperparameters of a convolutional neural network. The best particle fitness value was achieved by the APSO–CNN algorithm at 0.1431772 and by the APSO–WOA–CNN algorithm at 0.1319339 after iterative optimization. Thus, the APSO–WOA–CNN performed better than the APSO–CNN algorithm. We evaluated the APSO–WOA–CNN algorithm against APSO–CNN, SVM, and FNN. The results show that the APSO–WOA–CNN algorithm improves accuracy by 1.25%, average precision by 1%, the kappa coefficient by 11%, Hamming loss by 1.2%, and the Jaccard similarity coefficient by 2%, as compared to the APSO–CNN algorithm, and the APSO–CNN algorithm achieves the best performance, as compared to other algorithms.

### 6.1. Study limitations

Our research article has limitations. The proposed model is time consuming for optimization of the hyperparameters of a convolutional neural network.

## 7. Conclusions and future work

Until recently, intrusion detection systems have primarily used deep learning algorithms. A hybrid APSO–WOA–CNN algorithm was presented in this study. It detects multi-type attacks of IoT network attacks. With the proposed APSO-WOA optimization algorithm, the hyperparameters of a 1D CNN structural network can be optimized. In the first training stage of the CNN algorithm, the APSO–WOA optimization algorithm fitness value is the cross-entropy loss function value. The loss function measures the variance between the actual and expected values. When the variance degree is at its lowest, the model performs well. In the model training, the APSO–WOA–CNN algorithm had the best performance in comparison to the FNN algorithm, which used manual parameter settings. We compared our optimization algorithm to other optimization algorithms, including the APSO–CNN algorithm. The results show that the APSO–WOA–CNN algorithm improves accuracy by 1.25% and average precision by 1%, as compared to the APSO-CNN algorithm, because the APSO–CNN algorithm has the highest performance among the other algorithms. Thus, the APSO–WOA–CNN algorithm can detect multi-type network attacks better than the other algorithms.

In future work, we plan to use other optimization algorithms, such as the ant colony algorithm, genetic algorithm, artificial bee colony algorithm, Henry gas solubility algorithm, red fox, and grey wolf optimization algorithm to optimize the one-dimensional CNN’s structural parameters and to reduce the time complexity. Furthermore, more data preprocessing techniques will be included to filter and merge the network attack datasets and to choose the most effective features.
